# Major Factors for the Persistent Folding of Hybrid α, β, γ-Hybrid Peptides Into Hairpins

**DOI:** 10.3389/fchem.2020.530083

**Published:** 2020-09-29

**Authors:** Yulong Zhong, Quan Tang, Daniel P. Miller, Eva Zurek, Rui Liu, Zhong-Lin Lu, Bing Gong

**Affiliations:** ^1^Department of Chemistry, University at Buffalo, The State University of New York, Buffalo, NY, United States; ^2^College of Chemistry, Beijing Normal University, Beijing, China; ^3^Department of Chemistry, Hofstra University, Hempstead, NY, United States

**Keywords:** hybrid peptide, foldamer, hydrogen bond, β-turn, unnatural amino acid, β-hairpin

## Abstract

Factors responsible for the persistent adoption of hairpin conformations by hybrid oligopeptides, each having a central β/α dipeptide segment flanked by aromatic γ-amino acid (γAr) residues, are probed. Our recent studies revealed that tetrapeptide **1** and **2**, having central dipeptide segments consisting of β-alanine (β-Ala) and glycine (Gly), and L-β-homophenylalanine (L-β-homoPhe) and Gly residues, respectively, that are flanked by γAr residues, fold into well-defined, expanded β-turns with doubly H-bonded γAr residues. Replacing the γAr residues of **1** and **2** with L-Val and L-Leu residues results in tetrapetides **1′** and **2′** that fail to fold into defined conformations, which confirms the decisive role played by the H-bonded γAr residues in the promoting folding of **1** and **2**. Attaching L-Val and L-Leu residues to the termini of **1** affords hexapeptide **1a**. With an additional H-bond between its L-Val and L-Leu residues, peptide **1a** folds into a hairpin with higher stability than that of **1**, indicating that the expanded β-turn can nucleate and stabilize β-hairpin with longer β-strands. Attaching L-Val and L-Leu residues to the termini of **2** affords hexapeptide **2a**. Substituting the L-β-homoPhe residue of **2a** with a D-β-homoPhe residue gives hexapeptide **2b**. Surprisingly, hexapeptide **2a** fold into a hairpin showing the similar stability as those of tetrapeptides **1** and **2**. Hexapeptide **2b**, with its combination of a D-β-homoPhe residue and the L-Val/L-Leu pair, fold into a hairpin that is significantly more stable than the other hybrid peptides, demonstrating that a combination of hetero-chirality between the β-amino acid residue of the dipeptide loop and the α-amino acid residues of the β-strands enhances the stability of the resultant β-hairpin.

## Introduction

As a major class of protein secondary structure, reverse turns provide sites of chain reversal, which results in the globular character of a protein (Smith and Pease, 1980; Milner-White and Poet, 1987). Reverse turns include the widely occurring two-residue β-turns (Wilmot and Thornton, 1988), along with the less prevalent γ-turns (Némethy and Printz, 1972) and α-turns (Pavone et al., 1996). β-Turns and β-hairpins are frequently found in hairpin loops of globular proteins and play a key role in protein folding (Marcelino and Gierasch, 2008). The design of discrete β-hairpins relies on the availability of type II′ β-turn of D-Pro-Gly (Haque et al., 1996; Karle et al., 1996; Haque and Gellman, 1997; Espinosa and Gellman, 2000; Syud et al., 2001; Aravinda et al., 2004) segment and type I′ β-turns of Asn-Gly (de Alba et al., 1997; Maynard and Searle, 1997; Simpson et al., 2005) and Aib-D-Ala (Aravinda et al., 2002) segments. Hairpins and reverse turns including β-turns play crucial roles in initiating the folding of peptides and proteins (Jäger et al., 2001; Rotondi and Gierasch, 2003; Du et al., 2004; Marcelino and Gierasch, 2008), and also possess in important biological functions, for example, as epitopes in protein–protein (Ripoll, 1992; Wilson and Stanfield, 1994; DeLano et al., 2000; Tyndall et al., 2005; Shukla and Sasidhar, 2015) and protein–nucleic acid (Churchill and Suzuki, 1989; Erard et al., 1990; Maynard and Searle, 1997; Shi et al., 1998; Leon et al., 2008) interactions. Our recent studies (Zhang et al., 2019; Tang et al., 2020) led to the discovery of a series of expanded β-turns sharing a β/α loop, i.e., a central dipeptide segment consisting of a β and α amino acid residue that is flanked by doubly H-bonded aromatic γ-amino acid (γAr) residues. This expanded β-turn represents a surprisingly resilient turn motif that allows the incorporation of different α and β amino acid residues (Tang et al., 2020). It was found that introducing various α amino acid residues into the β/α dipeptide loop results in β-hairpins with the same or slightly lower stabilities, while incorporating β amino acid residues enhances the stabilities of the resultant β-hairpins. In this study, we explore the role of the γAr residues in the folding of this series of hybrid peptides. The effects of additional α-amino acid residues added to the N- and C-termini of the hybrid tetrapeptides to the stabilities of the resultant folded structures. The combinations of chirality between the β-amino acid residue in the dipeptide loop and the terminal α-amino acid residues are also examined for its influence on the folding of the corresponding hybrid peptides.

## Materials and Methods

### Chemistry

Reagents and solvents were purchased from commercial sources and used without further purification. Column chromatography was carried out on silica gel (300~400 mesh). ^1^H NMR spectra were recorded at 400 MHz and 600 MHz on a Bruker-400 spectrometer and JEOL-400 and 600 spectrometers at ambient temperature. ^13^C NMR spectra were measured at 100 MHz and 150 MHz on the same spectrometers. Chemical shifts are reported in parts per million downfield from TMS (tetramethylsilane). Coupling constants in ^1^H NMR are expressed in Hertz. Electrospray ionization high resolution mass spectra (ESI-HRMS) were acquired using a waters LCT Premier XE spectrometer (Waters, Milford, MA, USA).

### Computational Methods

The models for **2a** and **2b** were optimized with the revPBE-D3 (Zhang and Yang, 1998; Grimme et al., 2010) functional and dispersion correction using the Amsterdam Density Functional (ADF) (Fonseca Guerra et al., 1998; te Velde et al., 2001)^1^ software package. The triple-zeta with polarization functions (TZP) basis set was used while keeping the core 1s electrons fixed in the oxygen, nitrogen, and carbon atoms (van Lenthe and Baerends, 2003). The revPBE-D3 functional and dispersion correction were used due to its previous treatment of similar tetrapeptides (Zhang et al., 2019).

## Results and Discussion

### Synthesis

The synthesis of tetrapetides **1** and **2** has been reported by us (Zhang et al., 2019; Tang et al., 2020). Peptides **1′** and **2′** were prepared based on standard amide/peptide coupling.

**Figure 1:**
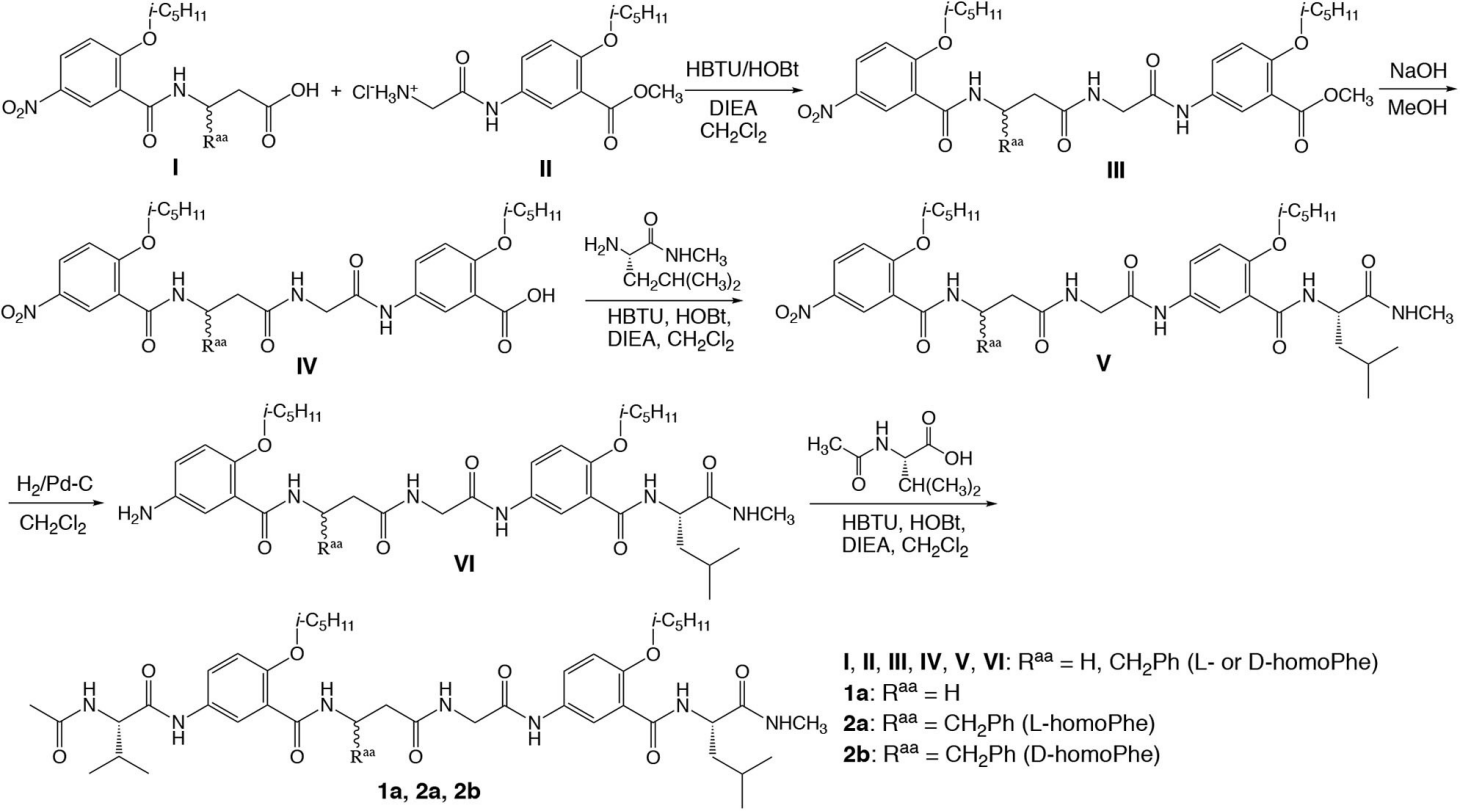
Synthesis of hybrid hexapeptides **1a**, **2a**, and **2b**.

Figure 1 shows the general steps and conditions for synthesizing hexapeptides **1a**, **2a**, and **2b**. Coupling **I** and **II**, which were prepared by coupling the methyl ester of D- or L-β-homo-phenyalanine with 2-isopentyloxy-5-nitrobenzoic acid, and Boc-protected glycine with the methyl ester of 5-amino-2-isopentyloxybenzoic acid, results in oligomer III. Hydrolyzing the methyl ester gives **IV**, which is coupled with the L-leucine derived amide to give **V**. Subjecting **V** to catalytic hydrogenation results in **VI**, followed by coupling with acetyl-L-valine to give peptides **1a**, **2a**, and **2b**. The detailed synthetic steps for preparing the intermediates and final products, along with the corresponding analytical data are included in the Supplementary Materials.

### The Critical Role of Aromatic γ-Amino Acid Residues

**Figure 2:**
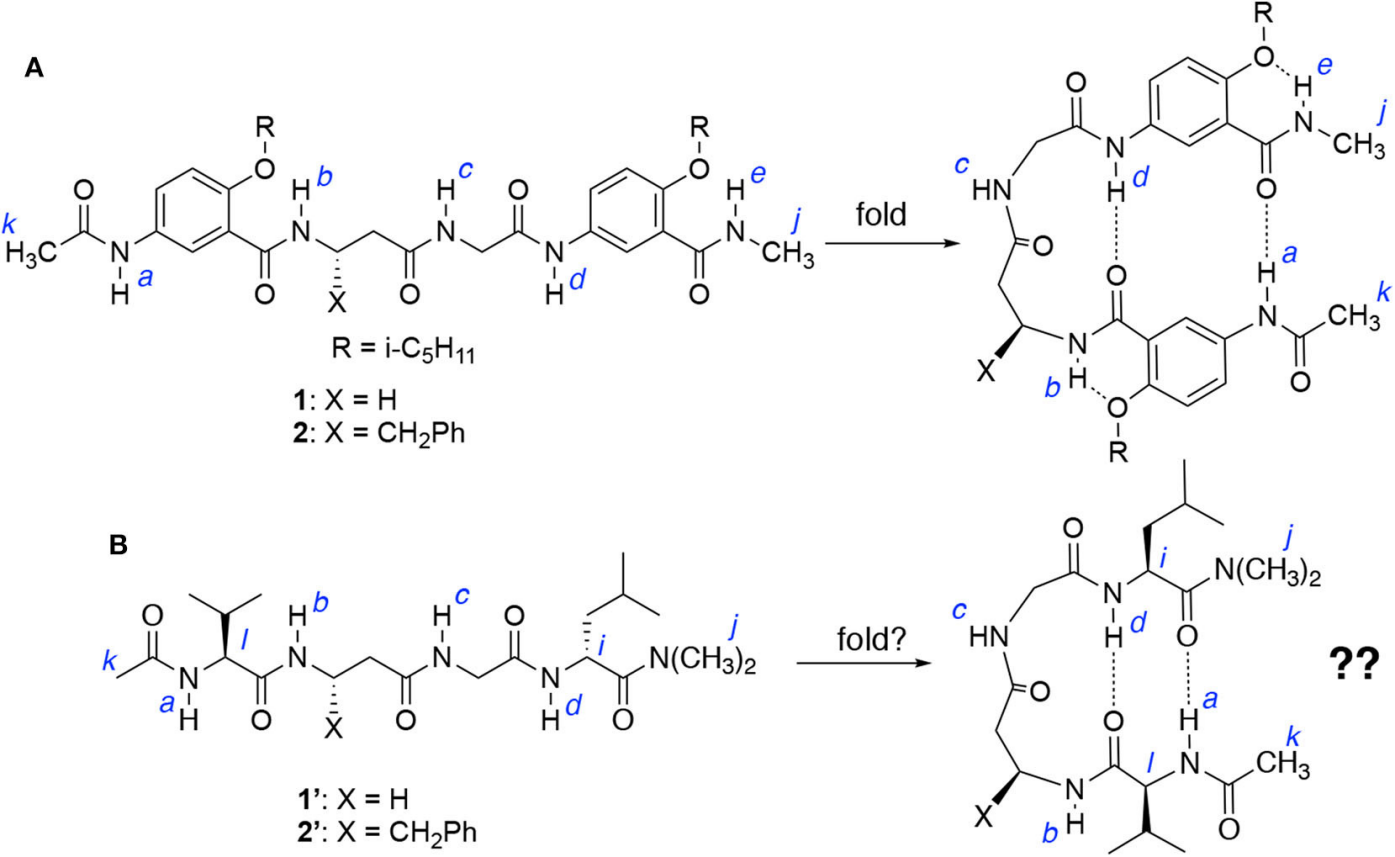
**(A)** Hybrid tetrapeptides **1** and **2** were found to fold into a hairpin conformation as shown. **(B)** Hybrid tetrapeptides **1′** and **2′** are designed to probe whether folded (hairpin) conformations could also be adopted.

Results from our recent study indicate that hybrid tetrapeptides **1** and **2**, along with eight other homologous hybrid peptides (Tang et al., 2020), persistently fold into a doubly H-bonded hairpin conformation containing an expanded β-turn that is very resilient toward incorporating different α- and β-amino acid residues into the central β/α dipeptide loop. Such a turn motif is capable of accommodating a variety of β/α dipeptide sequences that otherwise could not be introduced into a β-turn. For example (Figure 2A), glycine and β-alanine, i.e., homoglycine, two conformationally most flexible α- and β-amino acid residues, are found in tetrapeptide **1** which adopts a well-defined hairpin conformation. Replacing the β-alanine residue of **1** with other β-amino acid residues having side chains results in hybrid tetrapeptides such as **2** that adopts a hairpin conformation with enhanced stability.

Each of tetrapeptides **1** and **2**, like other hybrid tetrapeptides of this series, has two aromatic γ-amino acid (γAr) residues flanking the central β/α dipeptide segment. The vital role of the γAr residues in driving the folding of **1** and **2** is further demonstrated by examining the folding of tetrapeptides **1′** and **2′** (Figure 2B), which share the same β/α dipeptide segments with **1** and **2**, respectively, but the latter two have two α-amino acid residues, i.e., L-Val and L-Leu, that flank the β/α dipeptide segment. If folded, tetrapeptides **1′** and **2′** could also adopt doubly H-bonded hairpin conformations as shown in Figure 2B.

The ^1^H NMR spectra of **1** and **1′**, and **2** and **2′** recorded at 25 mM were compared to those recorded at 1 mM in CDCl_3_. Table 1 shows the difference in the chemical shifts (Δδ_NH_) of the amide protons of the four peptides at the two concentrations. In the folded conformations of **1** and **2**, protons *a* and *d*, like *b* and *e*, are intramolecularly H-bonded and exhibit either small upfield shifts or insignificant downfield shifts at high vs. low concentrations; while the signals of protons *c*, which are not intramolecularly H-bonded, shift noticeably downfield with increasing concentration (Tang et al., 2020). In contrast, all of the amide proton resonances of peptides **1′** and **2′** recorded at 25 mM show downfield shifts relative to those at 1 mM, with the signals of protons *a, c*, and *d* showing significant shifts, while those of protons *b* undergoing small shifts. These observations suggest that among the amide protons of **1′** and **2′**, only protons *b* are intramolecularly H-bonded. The fact that protons *a* and *d* of **1′** and **2′** are not intramolecularly H-bonded indicates that **1′** and **2′** do not fold into the hairpin conformation as shown in Figure 2B.

**Table 1:** Difference in the chemical shifts of amide protons at low and high concentrations*^a^*.

	**Δδ_NH_ (ppm)*^b^***				
**Entry**	**a**	**b**	**c**	**d**	**e**
**1**	−0.010	−0.026	0.662	−0.008	−0.043
**1′**	0.411	0.042	0.181	0.248	
**2**	−0.129	−0.057	0.536	0.046	−0.051
**2′**	0.376	0.065	0.306	0.367	

a *^1^H NMR spectra were recorded in CDCl_3_ (400 MHz, 298 K)*.

b *Δδ_NH_ = δ_(25mM)_–δ_(1 mM)_*.

The conformations of tetrapeptides **1′** and **2′** were examined with 2D (NOESY) spectroscopy (Supplementary Figures S4, S5). Except for NOEs between protons *b* and *c*, and *c* and *d*, the spectrum of **1′** reveals no NOEs between protons *a* and *d, i* and *l*, or *j* and *k*, which would exist if a hairpin conformation existed. The spectrum of **2′** contains an NOE between protons *b* and *c*, with no NOEs between protons *a* and *d, i* and *l*, or *j* and *k* being observed. The fact that only proton *b* of **1′** or **2′** is intramolecularly H-bonded, along with the absence of NOEs between other remote protons, suggests that **1′** and **2′**, being derived from replacing the aromatic γ-amino acid residues **1** and **2** with L-Val and L-Leu residues, cannot fold into hairpin conformations.

The above observations indicate that the doubly H-bonded γAr residues are indispensable in driving the folding of **1** and **2** into hairpin conformations. Without the γAr residues, peptides **1′** and **2′**, although capable of forming intramolecular H-bonds involving protons *a* and *d*, fail to adopt hairpin conformations. The critical role played by the γAr residues on stabilizing these novel hairpins relies on the effective H-bonding capabilities offered by these structural units (Gong, 2007), which provides the energetic driving force for the observed persistent folding of these hybrid peptides.

### Triply H-Bonded β-Hairpins: The Folding of Hexapeptide 1a

**Figure 3:**
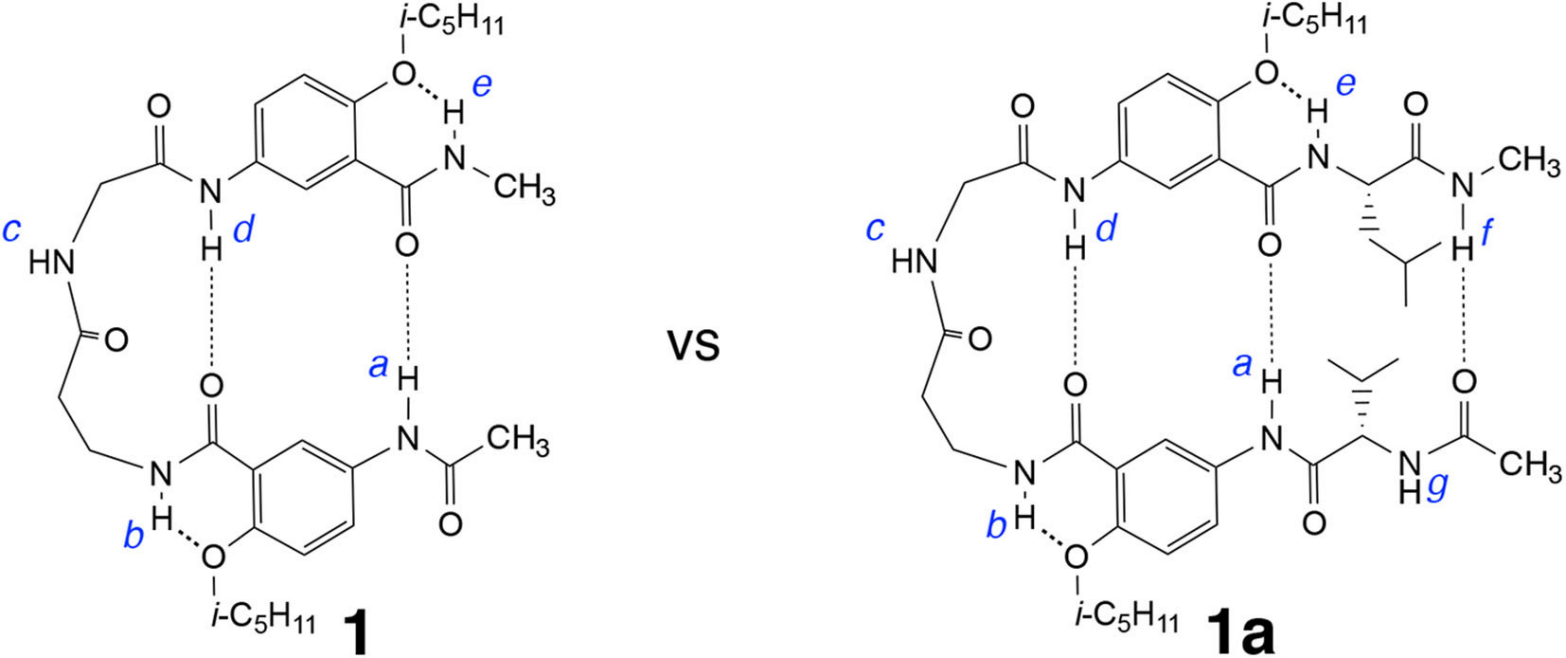
Hybrid hexapeptide **1a**, derived from tetrapeptide **1**, is expected to fold into a triply H-bonded hairpin conformation.

Comparing tetrapeptides **1′** with **1**, and **2′** with **2** revealed the critical importance of the doubly H-bonded γAr residues in ensuring the adoption of hairpin conformations by **1** and **2**. Attaching additional amino acid residues to **1** or **2** results in a longer peptide that may fold into a hairpin with an enhanced stability due to the energetic contribution of added H-bond(s). Hexapeptide **1a** (Figure 3), which is resulted from adding a pair of amino acid residues, L-Val and L-Leu, to the N and C termini of **1**, respectively, were examined and compared to tetrapeptide **1**.

The ^1^H NMR spectrum of **1a** recorded in CDCl_3_ at 25 °C contains well-dispersed signals (Supplementary Figure 1), indicating that **1a**, like **1**, exists as a single discrete species with a defined conformation. At 1 mM in CDCl_3_, the signals of protons *a* and *d* of **1a** appear at 10.06 and 9.53 ppm, respectively, while the same protons of **1** are found at 9.65 and 9.39 ppm. The downfield shifts of protons *a* and *d* of **1a** relative to those of **1** at the same concentration indicate that the H-bonds involving protons *a* and *d* of the former are stronger than those of the latter, which suggests that, hexapeptide **1a**, with one additional H-bond contributed by the L-Val/L-Leu pair, may very likely fold into a triply H-bonded conformation that is more stable than the doubly H-bonded hairpin of **1**.

Comparing the difference in the chemical shifts of amide protons at 25 mM and 1 mM in CDCl_3_ reveals that the amide proton resonances of **1a** follow the same trend as shown by those of **1** (Supplementary Table 1). The signal of proton *c* of **1** or **1a** undergoes the largest downfield shift (~0.7 ppm) upon increasing the concentration from 1 to 25 mM, suggesting that proton *c* of **1a**, like that of **1** (Tang et al., 2020), is intermolecularly H-bonded. In contrast, the signals of amide protons *a, b, d*, and *e* of **1a** exhibit very small (<0.01 ppm) upfield shifts, indicating that protons *a* and *d*, like *b* and *e*, are intramolecularly H-bonded. These observations suggest that hexapeptide **1a**, like tetrapeprtide **1**, folds into a hairpin conformation that is stabilized by H-bonds involving protons *a* and *d*, and further reinforced by a H-bond involving proton *f*. Indeed, proton *f* undergoes an upfield shift of 0.274 ppm from 1 to 25 mM, suggesting that it is intramolecularly H-bonded.

**Figure 4:**
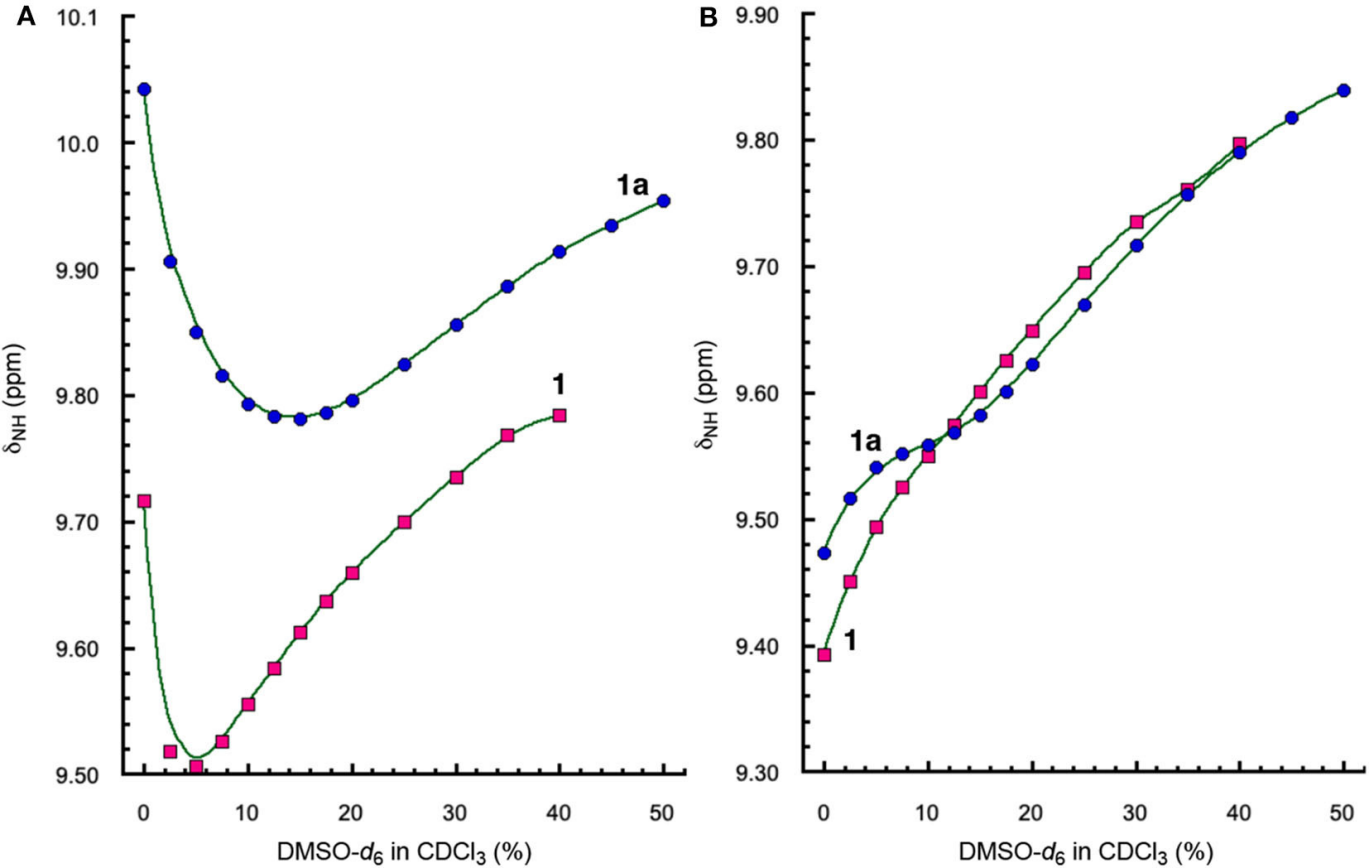
Plots of chemical shifts (δ_NH_) of amide protons **(A)** *a* and **(B)** *d*, of **1** (5 mM) and **1a** (5 mM) versus percent of DMSO-*d*_6_ in CDCl_3_.

The H-bonding interactions involving protons *a* and *d* were further examined by monitoring the chemical shifts of amide protons *a* and *d* of peptides **1** and **1a** in CDCl_3_ containing DMSO-*d*_6_. Interestingly, the signals of protons *a* and *d* shift differently with increasing ratios of DMSO (Figure 4). The resonances of protons *a* of both **1** and **1a** first shift upfield and then move downfield with as the ratio of DMSO increases (Figure 4A). In contrast, the signals of protons *d* of both **1** and **1a** show overall linear downfield shifts with increasing DMSO ratio (Figure 4B).

The different shifts of amide protons *a* and *d* toward increasing solvent polarity can be explained by the folded conformations of **1** and **1a** (Figure 3). Proton *d* is involved in an “intraturn”, N–H(*i*) → O=C(*i* −3) hydrogen bond that is part of the 11-atom, intramolecularly H-bonded ring that constitutes the expanded β-turn in the hairpin conformation of **1** or **1a**. Such a H-bond, with a H∙∙∙O distance of over 2.0 Å, is slightly longer and thus weaker than typical H-bonds. As a result, proton *d* of **1** or **1d** is more accessible to solvent molecules than proton *a*. With increasing proportion of DMSO, proton *d* becomes increasingly H-bonded with DMSO molecules, which results in the destabilization of the folded (hairpin) conformation. Such an overall destabilization of the hairpin conformation in turn weakens the H-bond involving proton *a*, which is reflected by the initial upfield shift of the signal of proton *a*. As the H-bond becomes further weakened, proton *a* also becomes more exposed to solvent molecules and engages in increasing H-bonding interaction with DMSO molecules, which leads to the downfield shift of its signal.

Comparing the shifts of amide protons *a* of **1** and **1a** reveals another interesting trend. As show in Figure 4A, the upfield shift shown by the signal of proton *a* of **1** is reversed at ~5% DMSO, while that of **1a** is reversed at 15% DMSO, indicating that proton *a* of **1a** is more resistant toward increasing solvent polarity than that of **1**, i.e., the H-bond involving proton *a* in **1a** is stronger than that in **1**. The stronger H-bond involving proton *a* of **1a** is mostly like due to the higher overall stability of the hairpin conformation of **1a** than that of **1**. The enhanced stability shown by the folded structure of **1a** can be explained by the energetic contribution of H-bonding involving proton *f* and the terminal amide C = O group.

**Figure 5:**
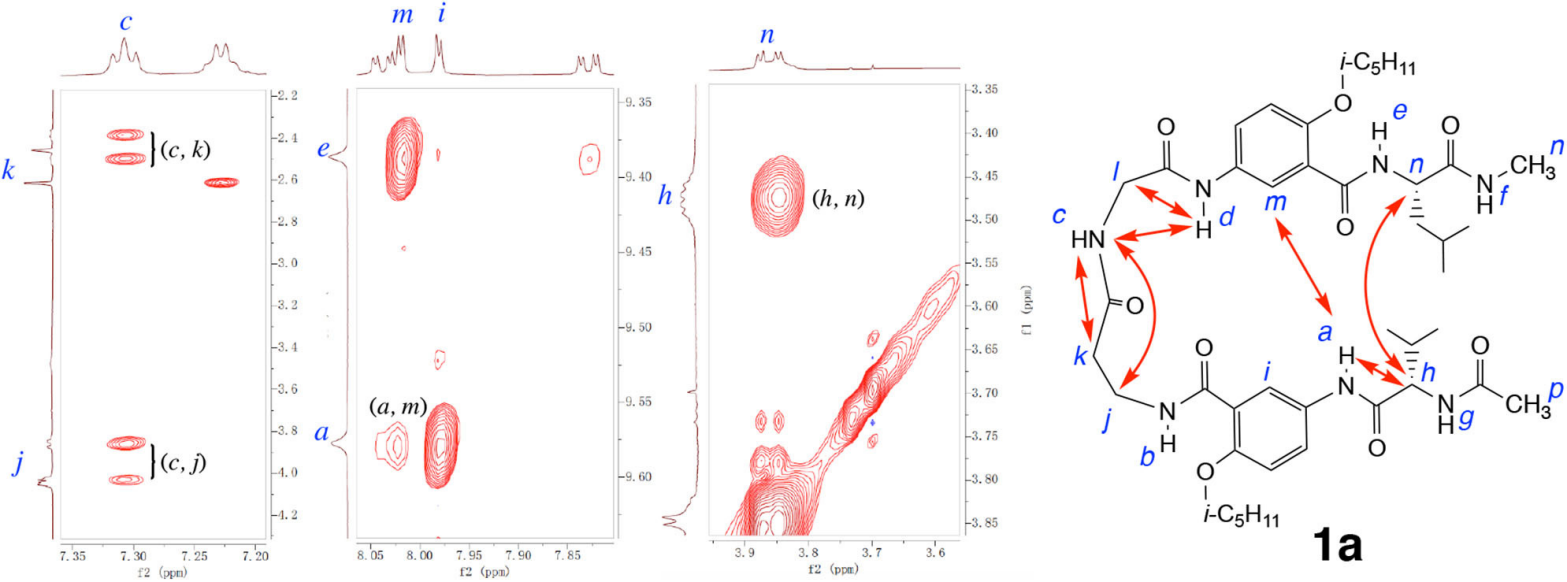
Partial NOESY spectra of hexapeptide **1a** (5 mM) in 1, 1, 2, 2-tetrachloroethane-*d*_2_ containing 5% DMSO-*d*_6_ (600 MHz, 298 K, mixing time: 300 ms). Major NOEs are indicated by double-headed arrows in the structure. Partial NOESY spectra showing NOEs between protons *a* and *h, c* and *d*, and *d* and *l* are included in the Supplementary Materials.

The folded conformation of hybrid peptide **1a** is confirmed by 2D NOESY spectra. As shown in Figure 5, the NOEs observed with **1a** include those between protons *a* and *m*, and *h* and *n*, which indicate the H-bonded alignment of the two aromatic γ-amino acid residues and the L-Val and L-Leu residues. In addition, NOEs between protons *c* and *j, c* and *k, c* and *d*, and *d* and *l* demonstrate the presence of a well-defined loop. These multiple NOEs confirm that hybrid peptide **1a** folds into a hairpin conformation similar to those observed with hybrid tetrapeptides **1** and **2** (Tang et al., 2020).

### Role of Residue Chirality on Hairpin Folding

**Figure 6:**
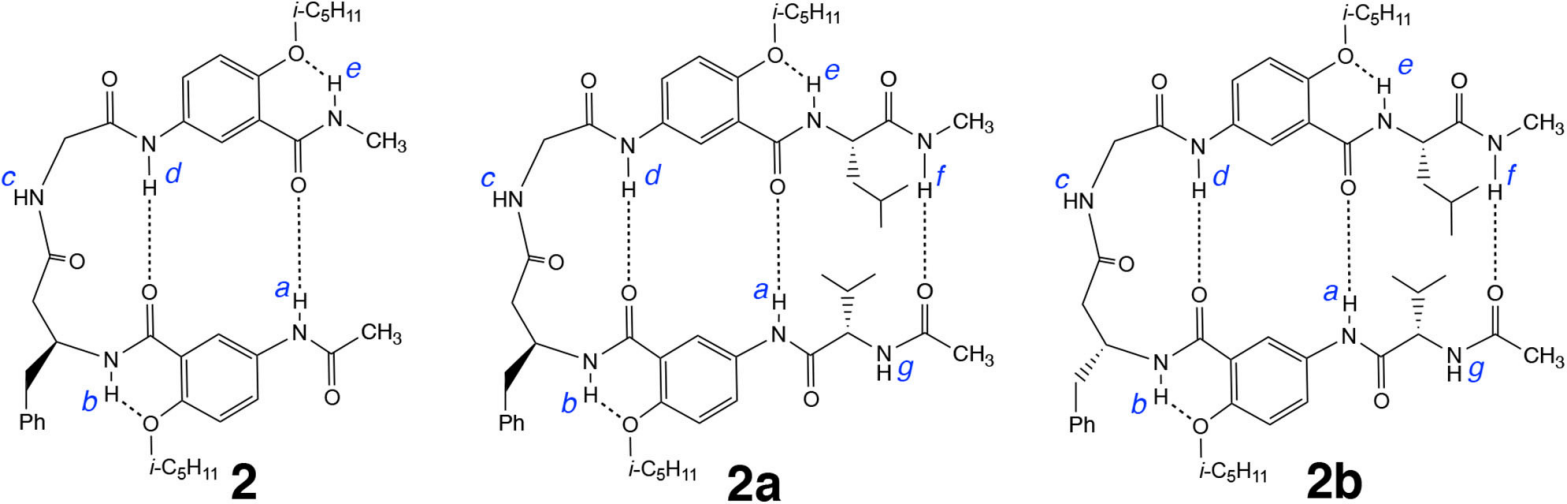
Hexapeptide **2a**, derived from tetrapeptide **2**, and hexapeptide **2b**, derived from **2a**, are expected to also adopt hairpin conformations.

Results from our recent study demonstrate that replacing the β-alanine residue of **1** with other β-amino acid residues such as β-homoPhe gives hybrid tetrapeptides including **2** that fold into expanded β-turns with enhanced stabilities (Tang et al., 2020). It is expected that the β-turn of **2**, like that of **1**, should also accommodate additional amino acid residues, resulting in longer β-hairpins. Attaching L-Val and L-Leu residues to the N and C termini of **2** results in hexapeptide **2a**. Replacing the L-β-homoPhe residue of **2a** with D-β-homoPhe gives hexapeptide **2b** (Figure 6). By comparing the folding of **2**, **2a**, and **2b**, and the stabilities of the folded structures, we intend to probe the compatibility of the chirality of the β-homoPhe residue in the dipeptide segment of the expanded β-turn with that of the two terminal L-α-amino acid residues.

Table 2 lists the difference in the chemical shifts of amide protons *a, b, c, d*, and *e* of peptides **2**, **2a**, and **2b**, along with the chemical shifts of amide protons *f* and *g* of **2a** and **2b**, measured at 25 mM and 1 mM. The amide protons of **2** and **2b** show the same overall change in their chemical shifts measured at high and low concentrations, suggesting that **2b**, like **2**, also folds into a well-defined hairpin conformation. In contrast, the amide proton resonances of **2a** show noticeable difference in that proton *c* does not undergo as large a downfield shift as those shown by protons *c* of **2** and **2b**. In addition, proton *d* of **2a** shows a significant downfield shift from 1 to 25 mM, which contrasts the negligible shifts observed with protons *d* of **2** and **2b**. These observations imply that, compared to those of **2** and **2b**, proton *d* of **2a** is more exposed and is available for intermolecular H-bonding, which in turn suggests that the expanded β-turn of **2a** involving H-bonded proton *d* might be partially twisted. Such a partial twisting or deformation may be resulted from the incompatibility of the L-homoPhe with the terminal L-Val and L-Leu residues of **2a**.

**Table 2:** Difference in the chemical shifts of amide protons at low and high concentrations*^a^*.

	**Δδ_NH_ (ppm)*^b^***						
**Entry**	**a**	**b**	**c**	**d**	**e**	**f**	**g**
**2**	−0.129	−0.057	0.536	0.046	−0.051	-	-
**2a**	0.039	−0.079	0.317	0.366	−0.090	−0.186	0.243
**2b**	−0.018	−0.007	0.666	−0.088	−0.002	−0.185	0.213

a *^1^H NMR spectra were recorded in CDCl_3_ (400 MHz, 298 K)*.

b *Δδ_NH_ = δ_(25mM)_–δ_(1 mM)_*.

**Figure 7:**
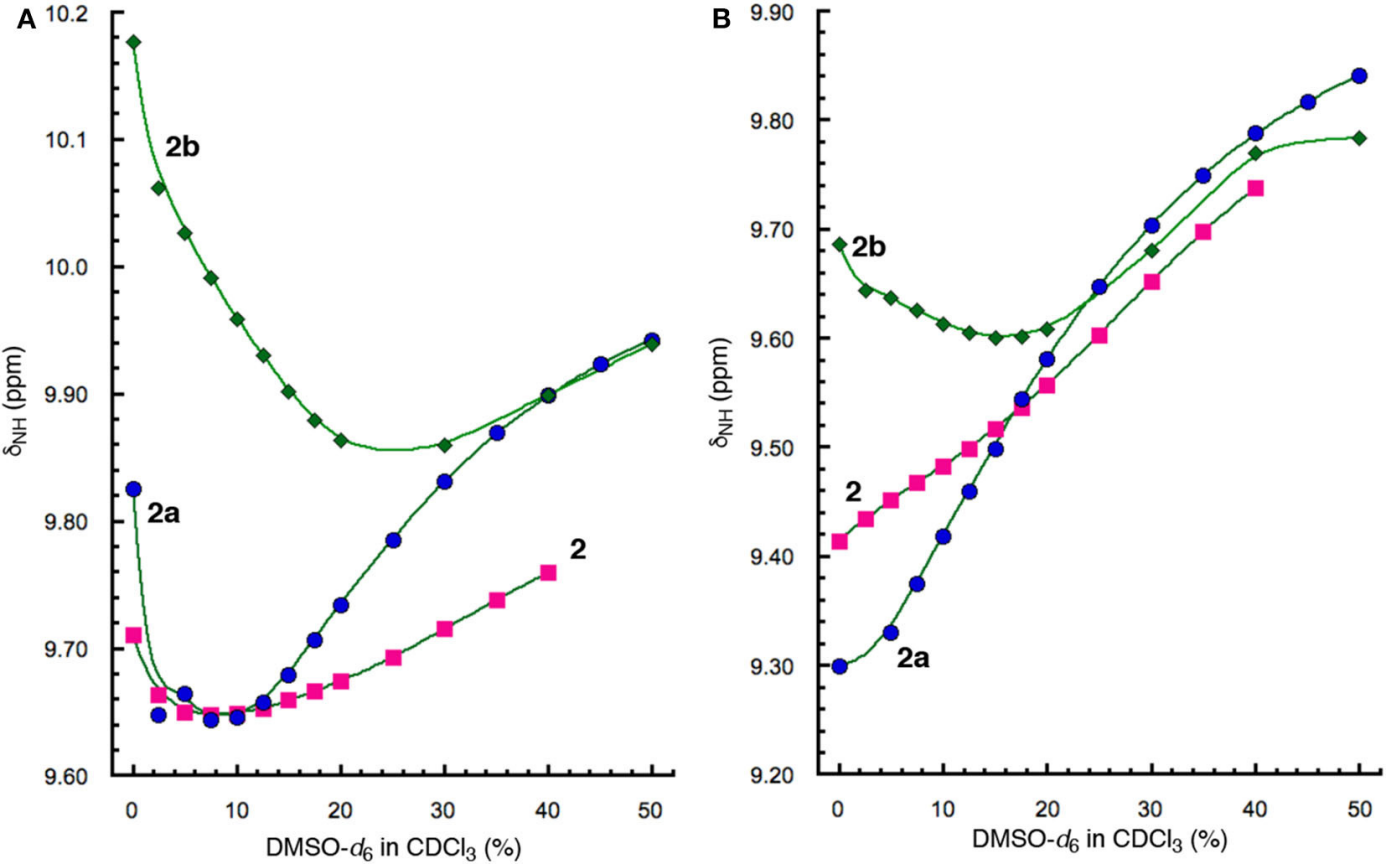
Plots of chemical shifts (δ_NH_) of amide protons **(A)** *a* and **(B)** *d*, of **2** (5 mM), **2a** (5 mM), and **2b** (5 mM) vs. percent of DMSO-*d*_6_ in CDCl_3_.

To assess the relative stabilities of the folded conformations of **2**, **2a**, and **2b**, the chemical shifts of protons *a* and *d* of these three hybrid peptides in CDCl_3_ containing DMSO-*d*_6_ were compared. Similar to what is observed with **1** and **1a** (Figure 4), the signals of protons *a* of **2**, **2a** and **2b** also first shift upfield with increasing proportion of DMSO, followed by shifting downfield as the ratio of DMSO further increases (Figure 7A). The resonances of protons *a* of the three peptides, however, undergo transitions from upfield to downfield shifts at different percent of DMSO, with peptide **2** showing its transition at ~6% DMSO, **2a** at ~8% DMSO, and **2b** at ~25% DMSO. Thus, proton *a* of **2b** is the least responsive toward increasing solvent polarity, which indicates that the folded conformation of **2b** is the most stable among those of the three peptides. The high stability of folded **2b** is also demonstrated by the upfied and then down field shift of proton *d* of this peptide (Figure 7B), which contrasts the consistent downfield shifts observed with the resonances of protons *d* of the other hybrid peptides, indicating that the H-bond involving proton *d* is greatly enhanced in the folded conformation of **2b**.

In contrast, the stability of folded hexapeptide **2a** is similar to that of tetrapeptide **2** and is much less stable than those of hexapeptides **2b** and **1a** (Figures 4A, 7A). The strong H-bonding and high stability of **2b**, and the much lower stability of **2a**, suggest that D-homoPhe residue in the H-bonded loop of **2b** is more compatible with the L-Val and L-Leu residues, while L-homoPhe of **2a** is much less compatible. Therefore, a hetero-chiral combination of the β-amino acid residue in the expanded β-turn and the α-amino acid residues in the β-strands seems to favor the nucleation and stabilization of β-hairpins. For example, an expanded β-strand with a D-β-amino acid residue should promote oligopeptides of L-α-amino acids to pair into a β-sheet.

**Figure 8:**
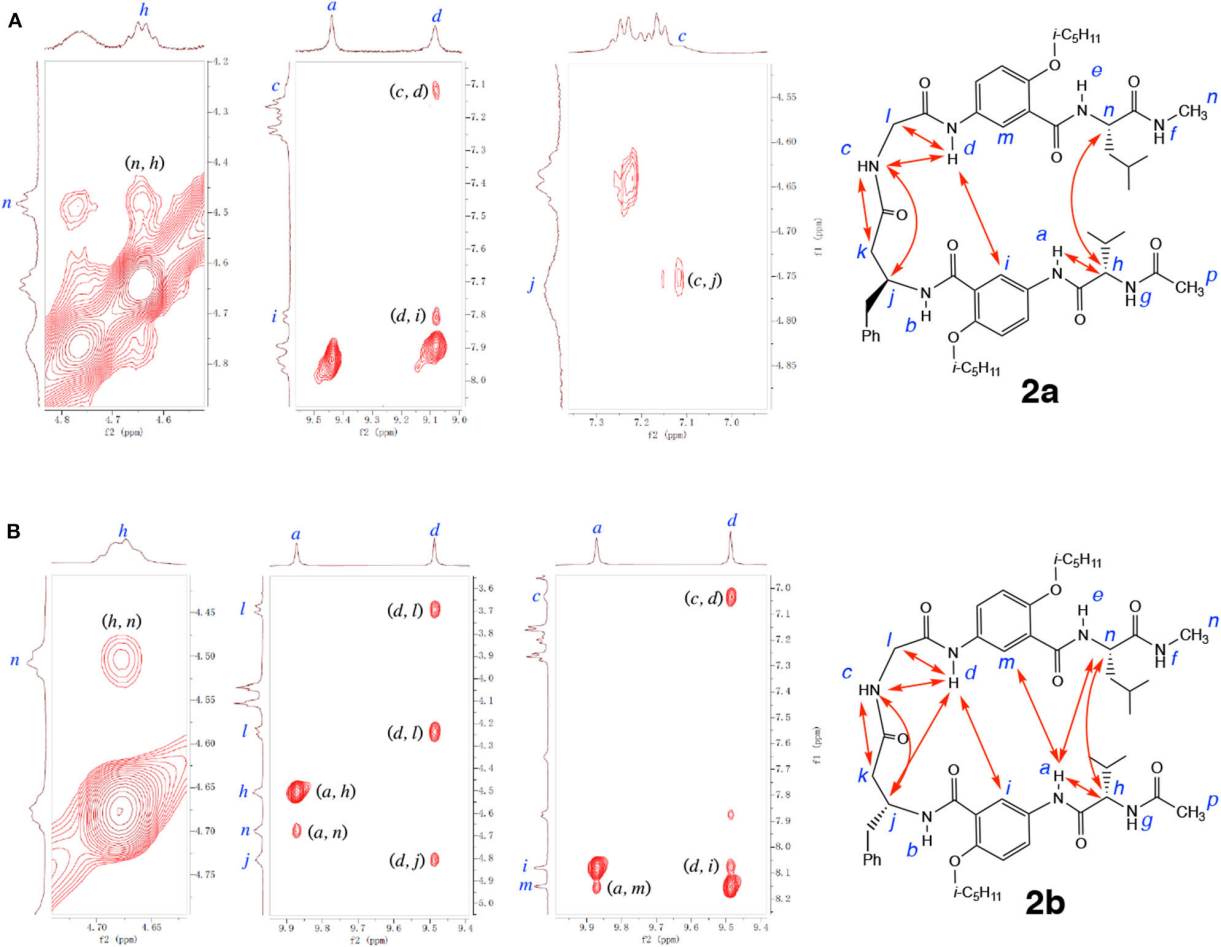
Partial NOESY spectra of **(A)** hexapeptide **2a** (5 mM), and **(B)** hexapeptide **2b** (5 mM) in 1, 1, 2, 2-tetrachloroethane-*d*_2_ containing 5% DMSO-*d*_6_ (600 MHz, 298 K, mixing time: 300 ms). Major NOEs are indicated by double-headed arrows in the structures. Partial NOESY spectra showing NOEs between protons *a* and *h, c* and *k*, and *d* and *l* of **2a**, and between protons *c* and *k*, and *c* and j of **2b**, are included in the Supplementary Materials.

NOESY spectra of **2a** and **2b** provide additional insights into the folding of these two hexapeptides. As shown in Figure 8A, the NOEs observed with **2a** include those between protons *d* and *i*, and *h* and *n*, which indicate the alignment of the two γAr residues and the L-Val and L-Leu residues. In addition, NOEs between protons *c* and *j, c* and *k, c* and *d*, and *d* and *l* demonstrate the presence of the H-bonded loop. The observed NOEs suggest that hybrid peptide **2a** adopts an overall hairpin conformation that includes a H-bonded loop along with the expected alignment of γAr and α-amino acid residues. In comparison to those of **2a**, NOEs with significantly stronger intensities are revealed by the NOESY spectrum of **2b** (Figure 8B). Strong NOEs that indicate the H-bonded alignment of the two γAr residues and the L-Val/L-Leu residues are clearly observed between protons *a* and *m, a* and *n, d* and *i*, and *h* and *n*. NOEs between protons *c* and *j, c* and *k, c* and *d, d* and *j*, and *d* and *l* are consistent with the presence of the 11-atom H-bonded ring that constitutes the expanded β-turn. The different numbers and strengths of the NOEs detected for **2a** and **2b** are consistent with the above conclusion on the different stabilities of the two folded structures. The numerous strong NOEs observed with **2b** is consistent with a compact, tightly folded conformation.

**Figure 9:**
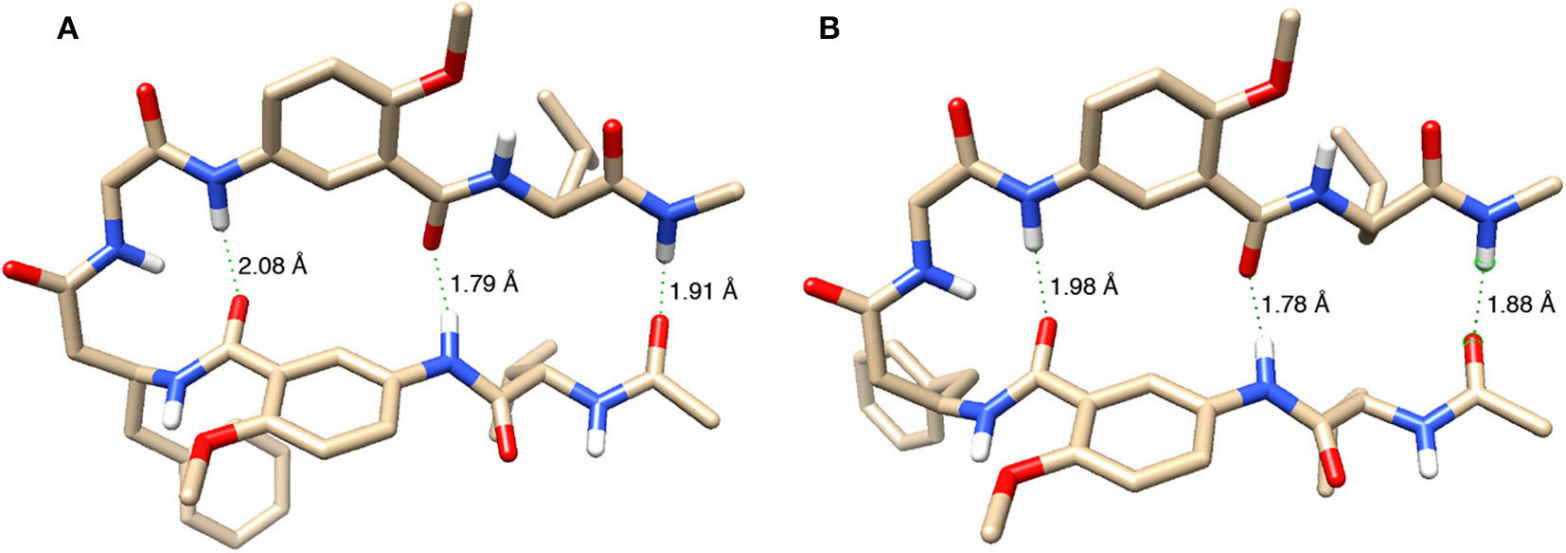
Energy-minimized structures of the hairpin conformations of **(A) 2a** and **(B) 2b**. Except for amide hydrogens, all other hydrogen atoms are removed for clarity. Three N-H∙∙∙O distances are indicated for each structure.

Finally, hexapeptides **2a** and **2b** were computationally optimized and compared. The optimized structures are shown in Figure 9. Modeling of **2a** with revPBE-D3 revealed N-H∙∙∙O hydrogen bond distances for protons *a, d*, and *f* to be 1.79, 2.08, and 1.91 Å, respectively. Measurements of the N-H∙∙∙O hydrogen bond distances in **2**b show that H-bonds involving protons *a, d*, and *f* shorten to 1.78, 1.98, and 1.88 Å, respectively, which suggest strengthened H-bonds. The strengthening of the hydrogen bonds in **2b** relative to **2a**, assessed through the N-H∙∙∙O hydrogen bond distances, coupled with **2a** having a larger buckling within the turnabout of the L-homoPhe residue relative to **2b** supports the experimental findings that **2b** holds a greater overall stability than **2a**.

## Conclusion

Results from this study have demonstrated that, by comparing with tetrapeptides consisting of α-amino acid residues that fail to fold into hairpin conformations, hybrid tetrapeptides sharing a general structure with a central β/α dipeptide segment flanked by doubly H-bonded γAr residues reliably fold into hairpins, which demonstrate the decisive role played by the γAr residues in driving the folding of these short peptides. Adding additional α-amino acid residues to the hybrid tetrapeptides results hexapeptides that fold into hairpins with enhanced stabilities, indicating that the expanded β-turns formed by the tetrapetides can effectively nucleate and stabilize longer hairpins. A combination of hetero-chirality between the β-amino acid residue, i.e., L- and D-homoPhe residues, in the dipeptide loop and the terminal α-amino acid residues, i.e., L-Val and L-Leu, strongly promotes the folding of the hexapeptides, based on which longer peptide strands should be aligned into defined β-sheets.

## Data Availability Statement

The raw data supporting the conclusions of this article will be made available by the authors, without undue reservation, to any qualified researcher.

## Author Contributions

The project was designed, coordinated, and supervised by BG with assistance from YZ. Synthesis of the hybrid peptides was performed by QT and YZ, under supervision of BG, Z-LL, and RL. The measurement of spectroscopic data was performed and analyzed by YZ and QT. The molecular modeling study was designed by DM and YZ, supervised by EZ and BG, and executed mainly by DM. BG analyzed and compiled the data and prepared the manuscript with support of YZ, and also of QT, DM, and EZ. The final manuscript was read and approved by all authors.

## Conflict of Interest

The authors declare that the research was conducted in the absence of any commercial or financial relationships that could be construed as a potential conflict of interest.

## Footnotes

^1^ADF, SCM, Theoretical Chemistry, Vrije Universiteit, Amsterdam, Netherlands. Available online at: http://www.scm.com.
