# Supplementary material for: Major Factors for the Persistent Folding of Hybrid α, β, γ-Hybrid Peptides Into Hairpins
Source: Front Chem. 2020 Sep 29;8:530083. doi: 10.3389/fchem.2020.530083 (PMC7550740; doi:10.3389/fchem.2020.530083)
Supplement: Supplementary file 1 [file Data_Sheet_1.PDF]

Supplementary Material

Major Factors for the Reliable Folding of Hybrid α, β, γ-Hybrid Peptides

Yulong Zhong[1†], Quan Tang[2†], Daniel P. Miller,[3] Eva Zurek[1], Rui Liu,[2] Zhong-Lin Lu,[2*] Bing Gong[1*]

[1]Department of Chemistry, University at Buffalo, The State University of New York, Buffalo, New York, USA

[2]College of Chemistry, Beijing Normal University, Beijing, China

[3]Department of Chemistry, Hofstra University, Hempstead, New York, USA.

[†]These authors have contributed equally to this work

*** Correspondence:**
Bing Gong
bgong@buffalo.edu

Zhong-lin Lu
luzl@bnu.edu.cn

1 Supplementary figures and tables

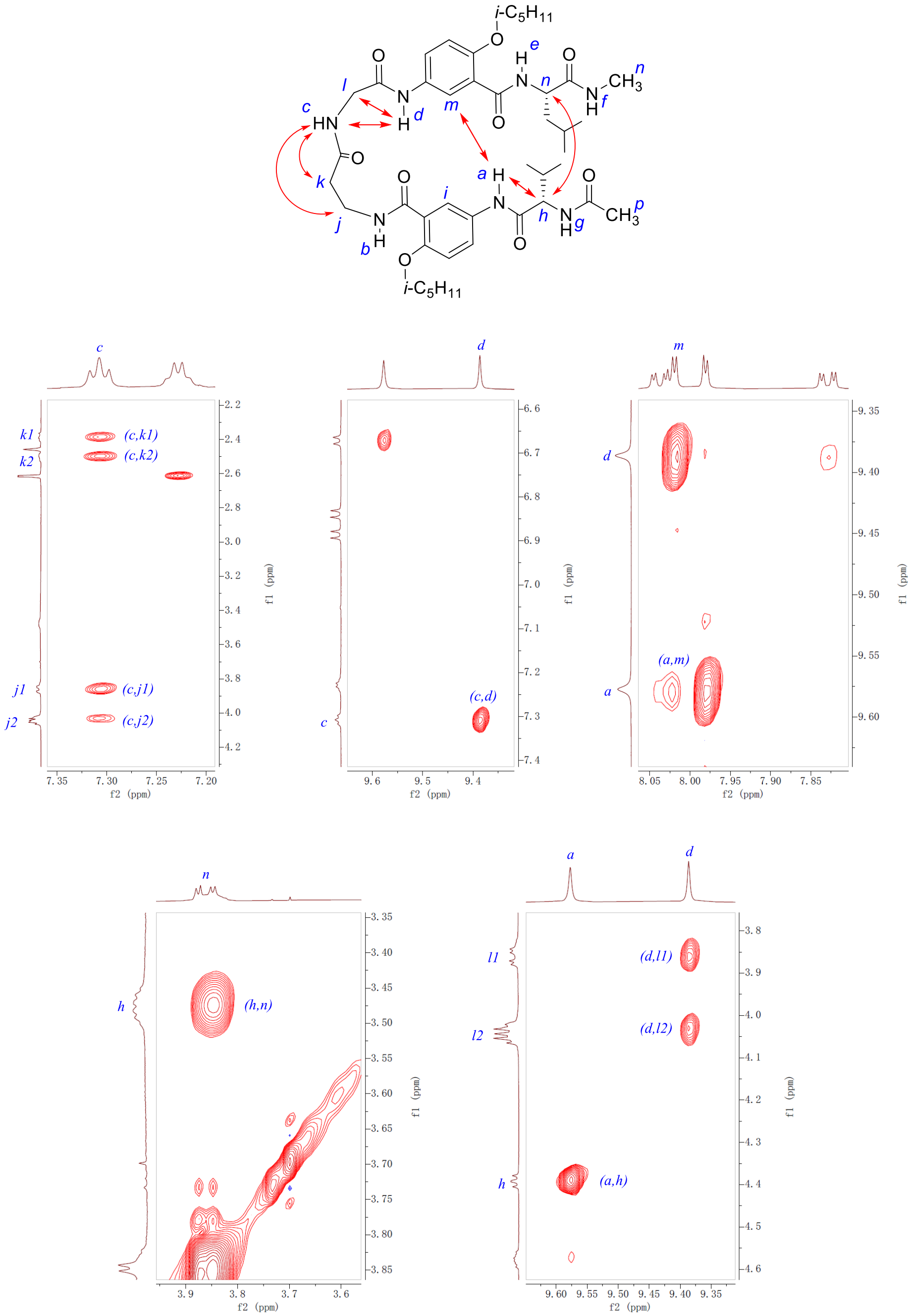

Supplementary Figure 1. Labeled structure of **1a** including observed NOE at 5 mM in mixed solvent of 1,1,2,2-Tetrachloroethane-d_2 with 5% of DMSO-d_6 (600 MHz, 298 K, 300 ms).

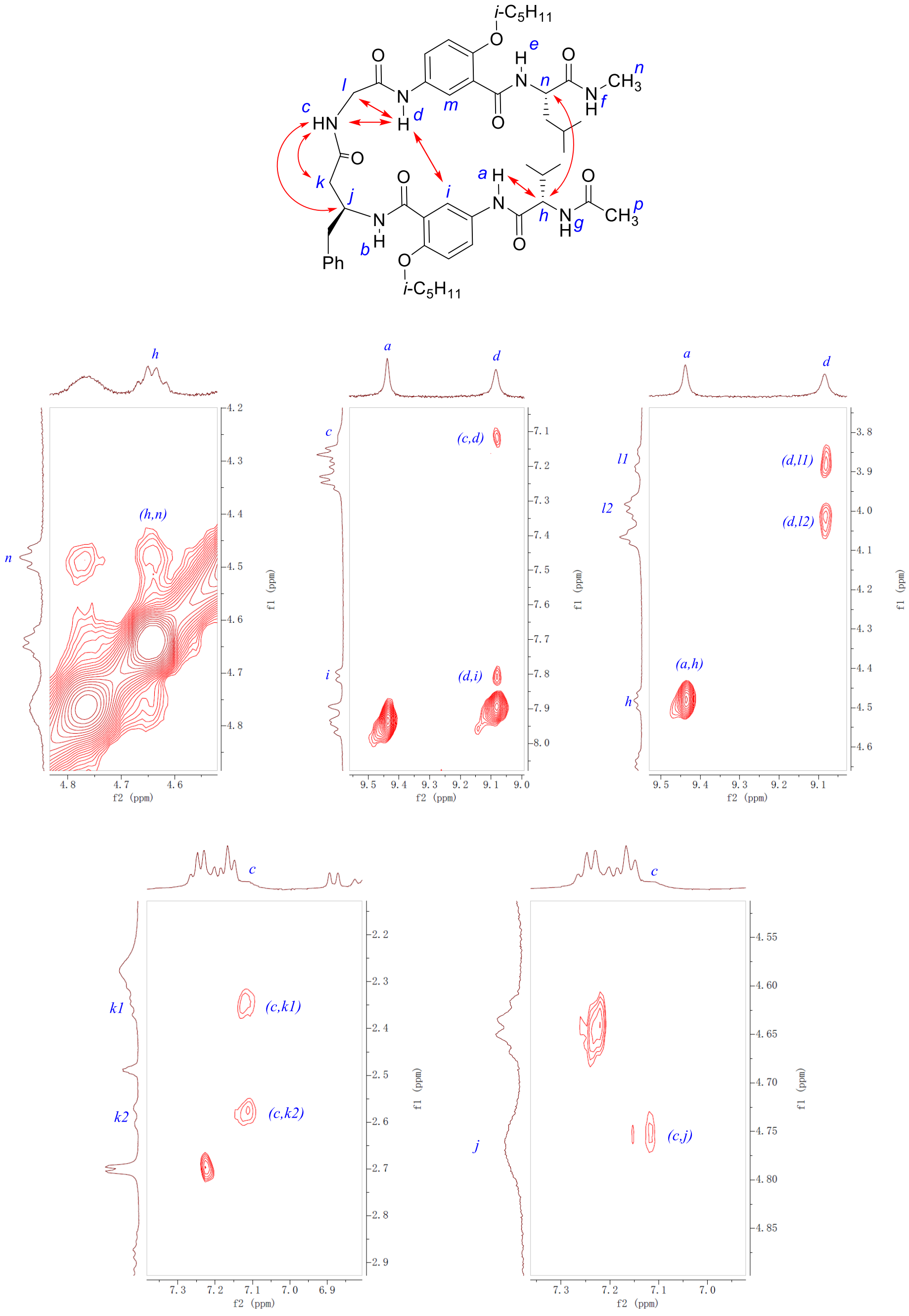

Supplementary Figure 2. Labeled structure of **2a** including observed NOE at 5 mM in mixed solvent of 1,1,2,2-Tetrachloroethane-d_2 with 5% of DMSO-d_6 (600 MHz, 298 K, 300 ms).

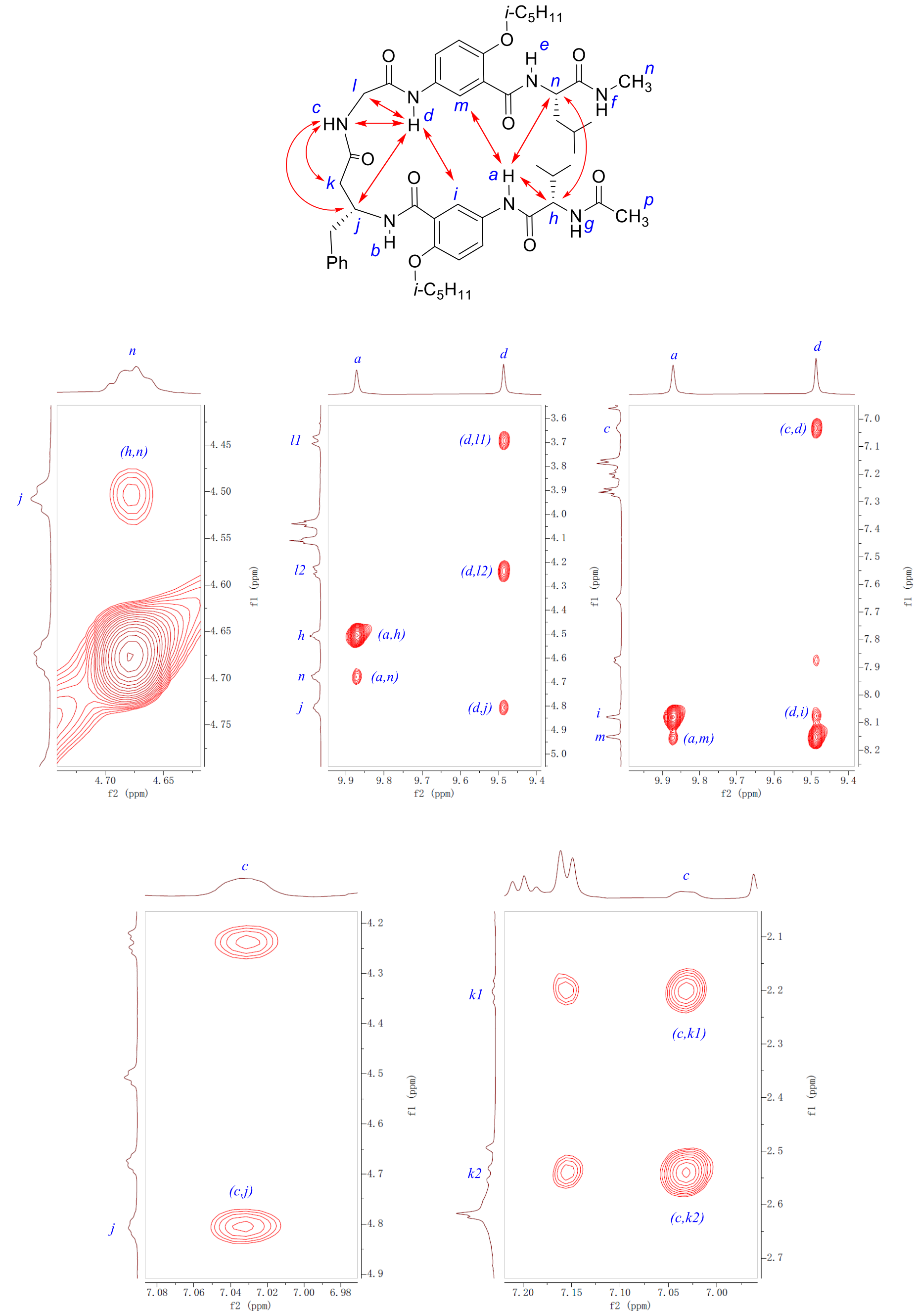

Supplementary Figure 3. Labeled structure of **2b** including observed NOE at 5 mM in mixed solvent of 1,1,2,2-Tetrachloroethane-d_2 with 5% of DMSO-d_6 (600 MHz, 298 K, 300 ms).

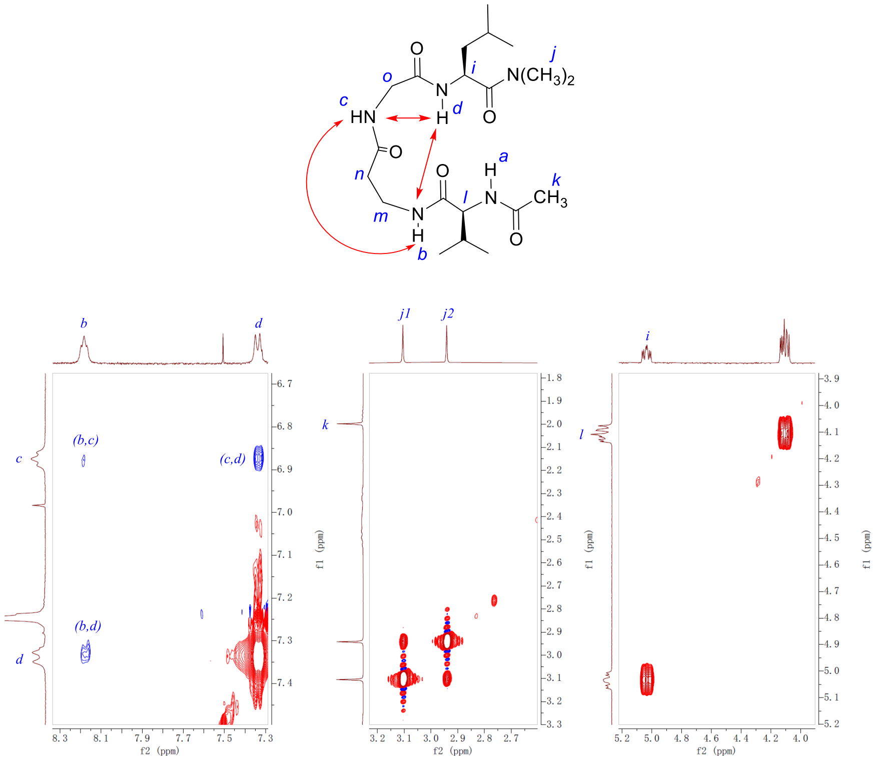

Supplementary Figure 4. Labeled structure of **1'** including observed NOE at 5 mM in $CDCl_3$ (400 MHz, 298 K, 300 ms).

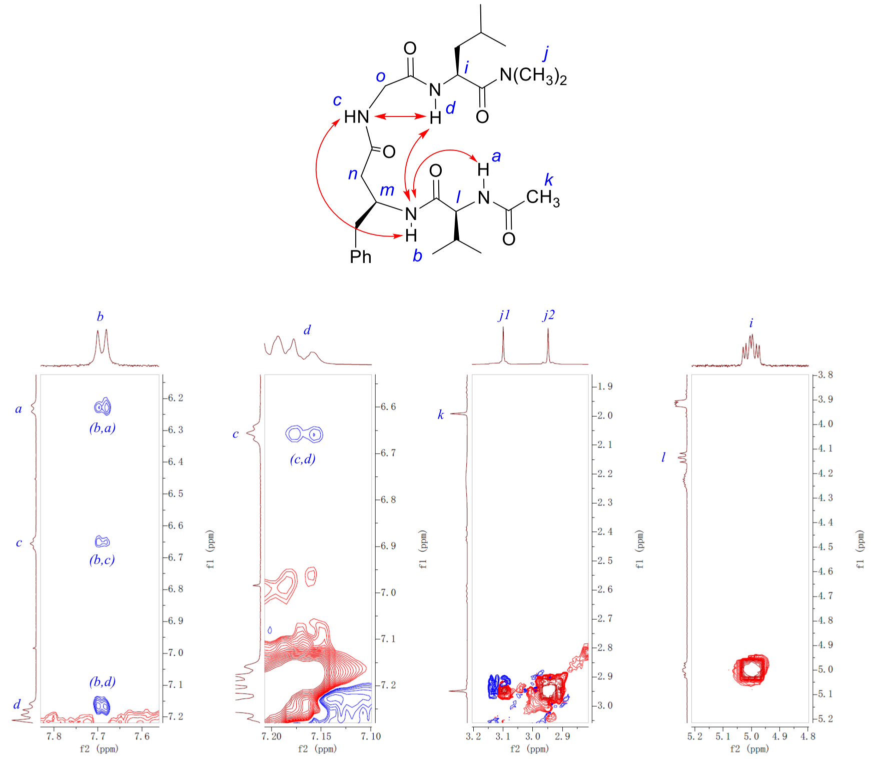

Supplementary Figure 5. Labeled structure of **2'** including observed NOE at 5 mM in $CDCl_3$ (400 MHz, 298 K, 300 ms).

Supplementary Table 1. Difference in the chemical shifts of amide protons of **1** and **1a** at low and high concentrations[a]

	$\Delta\delta_{NH}$ (ppm)[b]						
entry	a	b	c	d	e	f	g
**1**	-0.0095	-0.0258	0.6624	-0.0077	-0.0432	-	-
**1a**	-0.0689	-0.0834	0.7236	-0.0906	-0.0250	-0.2738	0.3469

[a] ^{1}H NMR spectra were recorded in $CDCl_3$ (400 MHz, 298 K). [b] $\Delta\delta_{NH} = \delta_{(25\ mM)} - \delta_{(1\ mM)}$.

2 Synthesis and Characterization

General remarks

Reagents and solvents were purchased from commercial sources and used without further purification. Compound **1'**, **2'** and **2b** are made based on standard amide/peptide coupling chemistry. Column chromatography was carried out on silica gel (300~400 mesh). ^{1}H NMR spectra were recorded at 400 MHz and 600 MHz on a Bruker-400 spectrometer and JEOL-400 and 600 spectrometers at ambient temperature. ^{13}C NMR spectra were measured at 100 MHz and 150 MHz on the same spectrometers. Chemical shifts are reported in parts per million downfield from TMS (tetramethylsilane). The ^{1}H NMR coupling constants are expressed in Hertz. Electrospray ionization high resolution mass spectra (ESI-HRMS) were recorded on a High resolution mass spectra acquired using a waters LCT Premier XE spectrometer (Waters, Milford, MA, USA)

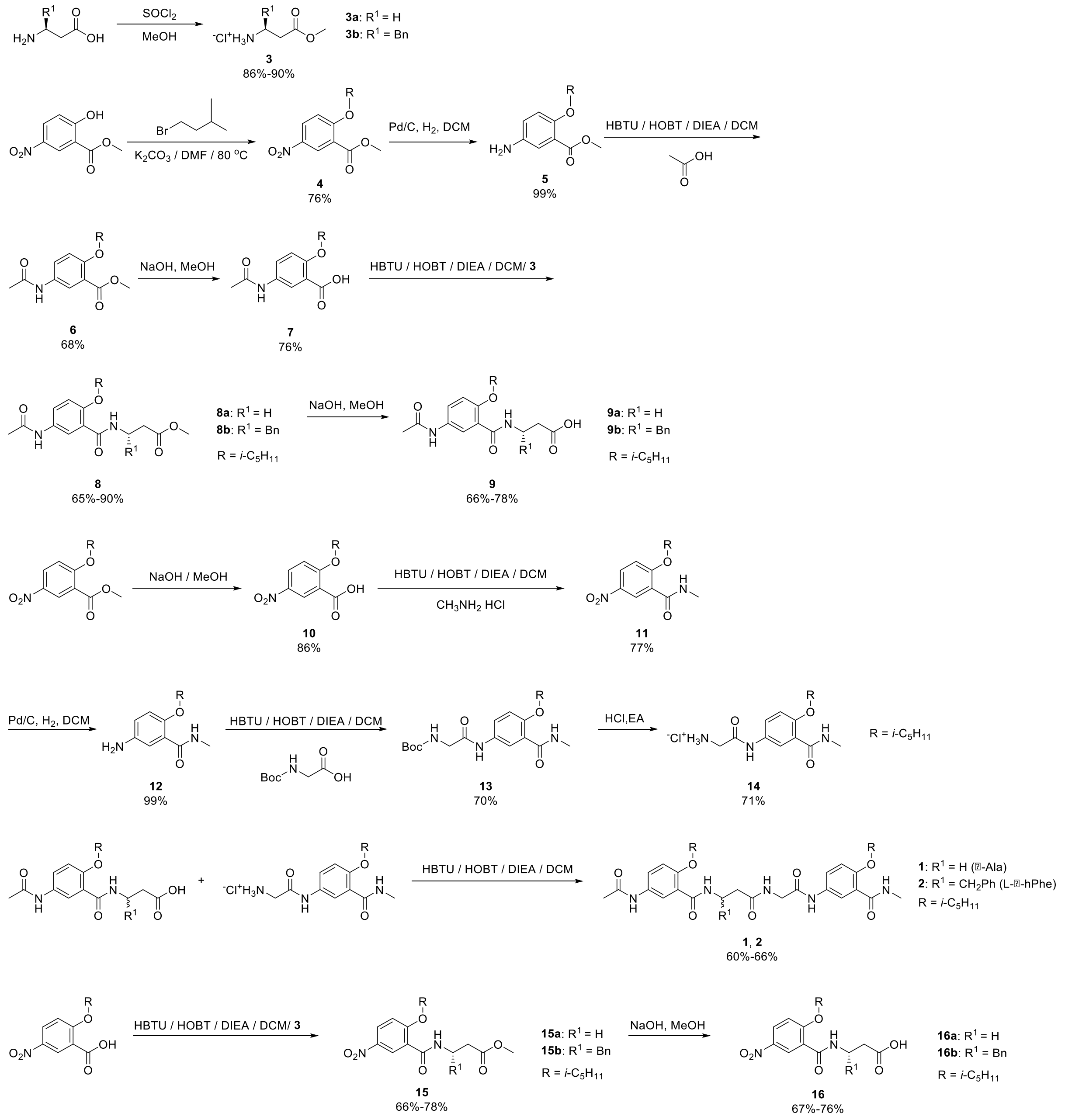

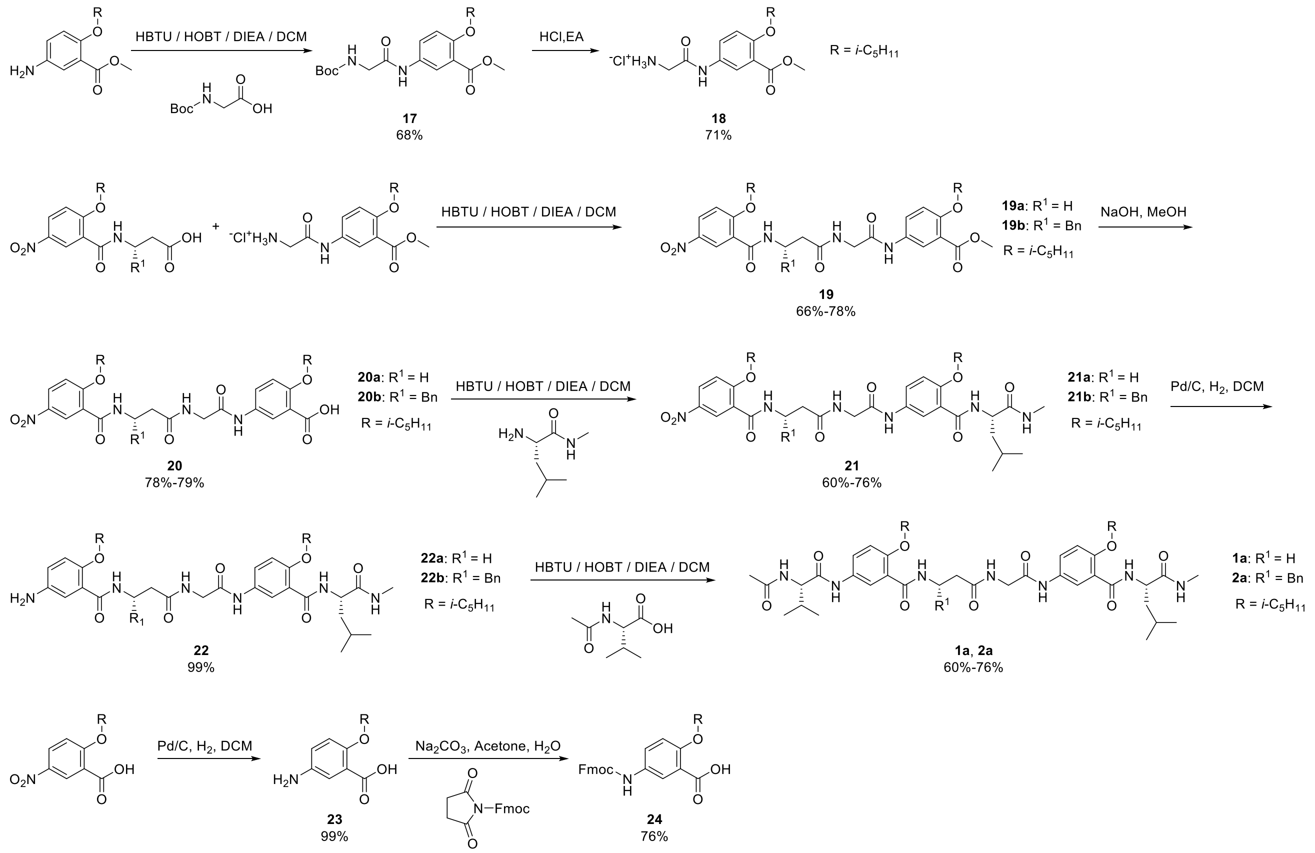

Compound **3a** and **3b** were synthesized according to previously reported procedures.[1]

Compound **4**. A mixture of methyl 2-hydroxy-5-nitrobenzoate (25.00 g, 126.81 mmol), K_2CO_3 (70.10 g, 507.24 mmol), 1-bromo-3-methylbutane (22.99 g, 152.17 mmol), KI (1.25 g, 7.53 mmol) and DMF (250 mL) was heated at 80 °C for 10 h. The K_2CO_3 was filtrated and washed by ethyl acetate. The filtrate and the extract were combined. After removing the solvent under reduced pressure, the remaining residue was dissolved in methylene chloride, washed with diluted HCl and brine, and dried over anhydrous Na_2SO_4. Removal of methylene chloride afforded the desired product as a yellow solid (25.74 g, 76%). ^{1}H NMR (400 MHz, DMSO-d_6) δ 8.43 (d, J = 3.0 Hz, 1H), 8.35 (dd, J = 9.2, 3.0 Hz, 1H), 7.37 (d, J = 9.2 Hz, 1H), 4.22 (t, J = 6.5 Hz, 2H), 3.33 (s, 3H), 1.90 – 1.76 (m, 1H), 1.69 – 1.60 (m, 2H), 0.93 (d, J = 6.6 Hz, 6H). ^{13}C NMR (151 MHz, $CDCl_3$) δ 163.80, 161.17, 141.91, 128.57, 127.94, 122.64, 112.53, 77.10, 68.91, 37.67, 26.80, 22.60. ESI-MS: calcd for $C_{13}H_{17}NO_5$ (M+Na$^+$) 290.10 found 290.13.

Compound **5**. Compound **4** (20.00 g, 74.87 mmol) was reduced by catalytic hydrogenation in dichloromethane (50 mL) at room temperature and atmospheric pressure, using Pd-C (0.20 g, 10%) as the catalyst. After all substrate was reduced, removal of the catalyst and solvent gave the product as a yellow oil. This compound was used without further purification.

Compound **6**. To a solution of acetic acid (5.40 g, 88.92 mmol) in 20 mL dichloromethane, HBTU (33.72 g, 88.92 mmol), HOBt (12.01 g, 88.92 mmol) and *N, N*-diisopropyl-ethylamin (22.99 g, 177.84 mmol) was added at 0 °C. 30 min later, the compound **5** (17.56 g, 74.09 mmol) in 15 mL dichloromethane was added to the mixture. Then the reaction was allowed to proceed for 8 h at room

temperature and inert atmosphere. The mixture was washed with diluted HCl and saturated brine, and solvent was removed in vacuum. Purification was accomplished by chromatography on silica gel using dichloromethane/MeOH to afford **6** as a white solid (14.06 g, 68%). ^{1}H NMR (600 MHz, $CDCl_3$) δ 7.77 (dd, *J* = 9.0, 2.8 Hz, 1H), 7.71 (d, *J* = 2.8 Hz, 1H), 7.24 (s, 1H), 6.93 (d, *J* = 9.0 Hz, 1H), 4.03 (t, *J* = 6.7 Hz, 2H), 3.86 (s, 3H) 2.16 (s, 3H), 1.91 – 1.81 (m, 1H), 1.75 – 1.65 (m, 2H), 0.95 (d, *J* = 6.7 Hz, 6H). ^{13}C NMR (151 MHz, $CDCl_3$) δ 168.42, 166.56, 155.66, 130.55, 126.00, 123.63, 120.53, 114.13, 67.99, 52.16, 37.98, 25.16, 24.47, 22.70. HRMS(ESI): calcd for $C_{15}H_{21}NO_4$: 280.1543 (M + H^+), found:280.1540 (M + H^+).

Compound **7**. The ester **6** (10.00 g, 35.82 mmol) was dissolved in MeOH (100 mL), to which 2 N NaOH (71.60 mL, 143.20 mmol) was added. The mixture was heated under reflux for 2 h. After removing the MeOH under vacuum, the aqueous layer was acidulated by addition of 3 N HCl to pH 2 followed by filtration to obtain a white solid (7.21 g, 76%).^{1}H NMR (600 MHz, $CDCl_3$) 11.27 (s, 1H), 8.94 (s, 1H), 8.39 (dd, *J* = 9.0, 2.8 Hz, 1H), 8.04 (d, *J* = 2.8 Hz, 1H), 7.01 (d, *J* = 9.1 Hz, 1H), 4.25 (t, *J* = 6.4 Hz, 2H), 2.23 (s, 3H), 1.86 – 1.74 (m, 3H), 0.98 (d, *J* = 6.2 Hz, 6H). ^{13}C NMR (151 MHz, $CDCl_3$) δ 169.53, 166.08, 153.74, 133.72, 127.26, 124.24, 117.28, 113.36, 69.24, 37.69, 25.18, 24.35, 22.54. HRMS(ESI): calcd for $C_{14}H_{19}NO_4$: 264.1241 (M - H^+), found: 264.1239 (M - H^+).

Compound **8a**. To a solution of compound **7** (2.00 g, 7.54 mmol) in 20 mL dichloromethane, HBTU (3.43 g, 9.05 mmol), HOBt (1.22 g, 9.05 mmol) and *N, N*-diisopropyl-ethylamin (2.43 g, 18.85 mmol) was added at 0 °C. 30 min later, the compound **3a** (1.05 g, 7.54 mmol) in 15 mL dichloromethane was added to the mixture. Then the reaction was allowed to proceed for 3 h at room temperature and inert atmosphere. The mixture was washed with diluted HCl and saturated brine, and solvent was removed in vacuum. The crude product was purified by column chromatography (petroleum ether/acetone = 5/1) to afford a white solid (2.38 g, 90%). ^{1}H NMR (400 MHz, DMSO-d_6) δ 9.91 (s, 1H), 8.29 (t, *J* = 5.8 Hz, 1H), 7.95 (d, *J* = 2.8 Hz, 1H), 7.75 (dd, *J* = 8.9, 2.8 Hz, 1H), 7.09 (d, *J* = 9.0 Hz, 1H), 4.08 (t, *J* = 6.6 Hz, 2H), 3.62 (s, 3H), 3.52 (q, *J* = 6.4 Hz, 2H), 2.58 (t, *J* = 6.5 Hz, 2H), 2.01 (s, 3H), 1.83 – 1.62 (m, 3H), 0.94 (d, *J* = 6.5 Hz, 6H). ^{13}C NMR (151 MHz, DMSO-d_6) δ 172.05, 167.93, 164.42, 152.15, 132.60, 123.14, 122.13, 121.50, 113.38, 67.44, 51.40, 37.14, 34.96, 33.55, 24.70, 23.76, 22.37. ESI-MS: calcd for $C_{18}H_{26}N_2O_5$ (M+Na^+) 373.17 found 373.27.

Compound **8b**. To a solution of compound **7** (2.00 g, 7.54 mmol) in 20 mL dichloromethane, HBTU (3.43 g, 9.05 mmol), HOBt (1.22 g, 9.05 mmol) and *N, N*-diisopropyl-ethylamin (2.43 g, 18.85 mmol) was added at 0 °C. 30 min later, the compound **3b** (1.73 g, 7.54 mmol) in 15 mL dichloromethane was added to the mixture. Then the reaction was allowed to proceed for 3 h at room temperature and inert atmosphere. The mixture was washed with diluted HCl and saturated brine. The combined organic layers were dried with sodium sulfate. The crude product was purified by column chromatography (petroleum ether/acetone = 5/1) to afford a white solid (2.16 g, 65%). ^{1}H NMR (600 MHz, DMSO-d_6) δ 9.90 (s, 1H), 8.28 (t, *J* = 5.8 Hz, 1H), 7.93 (d, *J* = 2.8 Hz, 1H), 7.71 (dd, *J* = 8.9, 2.8 Hz, 1H), 7.34 – 7.27 (m, 2H), 7.25 – 7.19 (m, 3H), 7.06 (d, *J* = 9.0 Hz, 1H), 4.05 (t, *J* = 6.6 Hz, 2H), 3.70 – 3.62 (m, 1H), 3.52 (s, 3H), 3.00 (dd, *J* = 13.6, 5.7 Hz, 1H), 2.77 (dd, *J* = 13.7, 8.4 Hz, 1H), 2.60 (dd, *J* = 16.8, 7.1 Hz, 1H), 2.52 (dd, *J* = 16.8, 5.5 Hz, 1H), 1.97 (s, 3H), 1.78 – 1.61 (m, 3H), 0.90 (d, *J* = 6.6 Hz, 6H). ^{13}C NMR (151 MHz, DMSO-d_6) δ 173.14, 170.16, 167.95, 164.26, 152.18, 135.79, 132.60, 129.41, 128.65, 127.05, 123.19, 122.03, 121.58, 113.41, 67.45, 51.77, 48.76, 38.02, 37.06, 35.82, 35.00, 33.79, 24.70, 23.75, 22.35. ESI-MS: calcd for $C_{25}H_{32}N_2O_5$ (M+H^+) 441.24 found 441.29.

Compound **9a**. The compound **8a** (2.0 g, 5.7 mmol) was dissolved in MeOH (100 mL), to which 2 N NaOH (11.4 mL, 22.8 mmol) was added. The mixture was heated under reflux for 2 h. After removing the MeOH under vacuum, the aqueous layer was acidulated by addition of 3 N HCl to pH 2 followed by filtration to obtain a white solid (1.3 g, 66%). ^{1}H NMR (600 MHz, DMSO-d_6) δ 12.33 (s, 1H), 9.93 (s, 1H), 8.33 (t, J = 5.8 Hz, 1H), 7.96 (d, J = 2.8 Hz, 1H), 7.76 (dd, J = 9.0, 2.8 Hz, 1H), 7.10 (d, J = 9.0 Hz, 1H), 4.09 (t, J = 6.6 Hz, 2H), 3.48 (q, J = 6.3 Hz, 2H), 2.48 (d, J = 6.5 Hz, 2H), 2.00 (s, 3H), 1.81 - 1.71 (m, 1H), 1.71 - 1.61 (m, 2H), 0.93 (d, J = 6.6 Hz, 6H). ^{13}C NMR (151 MHz, DMSO-d_6) δ 173.22, 167.97, 164.26, 152.22, 132.62, 123.20, 121.99, 121.59, 113.41, 67.45, 37.07, 35.01, 33.81, 24.73, 23.80, 22.38. HRMS(ESI): calcd for $C_{17}H_{24}N_2O_5$: 335.1612 (M - H^+), found: 335.1610 (M - H^+).

Compound **9b**. The compound **8b** (2.0 g, 4.5 mmol) was dissolved in MeOH (100 mL), to which 2 N NaOH (9.1 mL, 18.2 mmol) was added. The mixture was heated under reflux for 2 h. After removing the MeOH under vacuum, the aqueous layer was acidulated by addition of 3 N HCl to pH 2 followed by filtration to obtain a white solid. The solid was washed by methanol to get a white solid (1.5 g, 78%). ^{1}H NMR (400 MHz, DMSO-d_6) δ 8.37 (d, J = 3.0 Hz, 1H), 8.28 (dd, J = 9.2, 3.0 Hz, 1H), 8.26 (s, 1H), 8.24 (s, 1H), 7.34 (d, J = 9.2 Hz, 1H), 7.29 – 7.22 (m, 2H), 7.20 – 7.14 (m, 3H), 4.49 (q, J = 6.9 Hz, 1H), 4.20 (t, J = 6.5 Hz, 2H), 2.91 – 2.79 (m, 2H), 2.53 – 2.49 (m, 1H), 2.40 (m, 1H), 2.18 (s, 3H), 1.74 – 1.45 (m, 3H), 0.86 (t, J = 6.3 Hz, 6H). ^{13}C NMR (151 MHz, DMSO-d_6) δ 172.42, 172.03, 162.49, 161.16, 140.40, 138.12, 129.17, 128.30, 127.61, 126.38, 125.81, 123.94, 113.82, 68.36, 48.60, 47.68, 36.69, 24.43, 22.31, 22.23, 22.01, 21.07. ESI-MS: calcd for $C_{24}H_{30}N_2O_5$ (M+Na^+) 449.20 found 449.95.

Compound **10**. were synthesized according to previously reported procedures.[2]

Compound **11**. To a solution of compound **10** (2.00 g, 7.91 mmol) in 20 mL dichloromethane, HBTU (3.60 g, 9.49 mmol), HOBt (1.28 g, 9.49 mmol) and *N, N*-diisopropyl-ethylamin (2.45 g, 18.98 mmol) was added at 0 °C. 30 min later, methylamine hydrochloride (0.53 g, 7.91 mmol) in 15 mL dichloromethane was added to the mixture. Then the reaction was allowed to proceed for 3 h at room temperature and inert atmosphere. The mixture was washed with diluted HCl and saturated brine, and solvent was removed in vacuum. The crude product was purified by column chromatography (petroleum ether/acetone = 6/1) to afford a white solid (1.45 g, 69%). ^{1}H NMR (600 MHz, $CDCl_3$) δ 9.10 (d, J = 3.0 Hz, 1H), 8.30 (dd, J = 9.1, 3.0 Hz, 1H), 7.74 (s, 1H), 7.07 (d, J = 9.1 Hz, 1H), 4.27 (t, J = 6.3 Hz, 2H), 3.03 (d, J = 4.8 Hz, 3H), 1.96 – 1.76 (m, 3H), 1.03 (d, J = 6.4 Hz, 6H). ^{13}C NMR (151 MHz, $CDCl_3$) δ 163.86, 161.23, 141.97, 128.63, 128.00, 122.70, 112.59, 68.97, 37.73, 26.86, 25.54, 22.66. HRMS(ESI): calcd for $C_{13}H_{18}N_2O_4$: 267.1339 (M + H^+), found 267.1341 (M + H^+).

Compound **12**. Compound **11** (3.00 g, 11.28 mmol) was reduced by catalytic hydrogenation in dichloromethane (50 mL) at room temperature and atmospheric pressure, using Pd-C (0.30 g, 10%) as the catalyst. After all substrate was reduced, removal of the catalyst and solvent gave the product as yellow oil. This compound was used without further purification.

Compound **13**. To a solution of Boc-glycine (2.36 g, 13.47 mmol) in 30 mL dichloromethane, HBTU (5.11 g, 13.47 mmol), HOBt (1.82 g, 13.47 mmol) and *N, N*-diisopropyl- ethylamine (3.63 g, 28.06 mmol) was added at 0 °C. 30 min later, the compound **12** (2.65 g, 11.22 mmol) in 15 mL dichloromethane was added to the mixture. Then the reaction was allowed to proceed for 8 h at room temperature and inert atmosphere. The mixture was washed with diluted HCl and saturated brine, and

solvent was removed in vacuum. Purification by flash column chromatography (petroleum ether/ethyl acetate = 2/1) to afford a white solid (3.09 g, 70%). ^{1}H NMR (400 MHz, DMSO-d_6) δ 9.90 (s, 1H), 8.16 – 8.02 (m, 1H), 8.01 – 7.88 (m, 1H), 7.74 (d, *J* = 8.9 Hz, 1H), 7.08 (d, *J* = 9.0 Hz, 1H), 7.01 – 6.98 (m, 1H), 4.08 (t, *J* = 6.7 Hz, 2H), 3.71 (d, *J* = 6.1 Hz, 2H), 2.82 (d, *J* = 4.7 Hz, 3H), 1.78 – 1.60 (m, 3H), 1.39 (s, 9H), 0.92 (d, *J* = 6.6 Hz, 6H). ^{13}C NMR (101 MHz, DMSO-d_6) δ 168.00, 165.20, 155.97, 152.20, 132.21, 122.99, 121.60, 113.45, 78.08, 67.45, 43.76, 37.24, 28.22, 26.17, 24.87, 22.43. ESI-MS: calcd for $C_{20}H_{31}N_3O_5$ (M+Na$^+$) 416.47 found 416.08.

Compound **14**. Compound **13** (3.93 g, 10.00 mmol) was treated with hydrochloric solution of ethyl acetic (2 N, 30 mL), then the mixture was stirred at room temperature for 1 h. Removed the solvent to obtain compound **14** as a white solid.white solid (2.34 g, 71%). ^{1}H NMR (600 MHz, DMSO-d_6) δ 10.65 (s, 1H), 8.20 (s, 3H), 8.07 (q, *J* = 4.7 Hz, 1H), 7.96 (d, *J* = 2.8 Hz, 1H), 7.71 (dd, *J* = 8.9, 2.8 Hz, 1H), 7.15 (d, *J* = 9.0 Hz, 1H), 4.10 (t, *J* = 6.6 Hz, 2H), 3.75 (s, 2H), 2.80 (d, *J* = 4.7 Hz, 3H), 1.80 – 1.70 (m, 1H), 1.70 – 1.60 (m, 2H), 0.94 (d, *J* = 6.6 Hz, 6H). ^{13}C NMR (151 MHz, DMSO-d_6) δ 165.00, 164.47, 152.51, 131.37, 123.28, 122.92, 121.52, 113.64, 67.43, 40.89, 37.15, 26.19, 24.82, 22.45. HRMS(ESI): calcd for $C_{15}H_{23}N_3O_3$: 294.1812 (M + H$^+$), found: 294.1810 (M + H$^+$).

General procedure for the synthesis of compounds 1 and 2. To a solution of compound **9** (1.12 mmol) in 30 mL dichloromethane, HBTU (511 mg, 1.35 mmol), HOBt (182 mg, 1.35 mmol) and *N, N*-diisopropyl- ethylamine (363 mg, 2.81 mmol) was added at 0 °C. 30 min later, the compound **14** (1.12 mmol) in 15 mL dichloromethane was added to the mixture. Then the reaction was allowed to proceed for 6 h at room temperature and inert atmosphere. The mixture was washed with diluted HCl and saturated brine, and solvent was removed in vacuum. The crude product was purified by column chromatography (petroleum ether/ acetone = 2/1).

Compound **1**. White solid (412 mg, 60%). ^{1}H NMR (400 MHz, $CDCl_3$) δ 9.68 (s, 1H), 9.38 (s, 1H), 8.68 (t, *J* = 6.0 Hz, 1H), 8.33 (dd, *J* = 9.0, 2.4 Hz, 1H), 8.14 (q, *J* = 5.0 Hz, 1H), 8.08 – 8.04 (m, 3H), 6.97 (t, *J* = 5.7 Hz, 1H), 6.93 (p, *J* = 9.6 Hz, 1H), 6.80 (d, *J* = 9.0 Hz, 1H), 4.17 – 4.06 (m, 6H), 3.82 – 3.74 (m, 2H), 2.98 (d, *J* = 4.7 Hz, 3H), 2.60 – 2.51 (m, 2H), 2.17 (s, 3H), 1.87 – 1.74 (m, 6H), 1.01 (d, *J* = 5.7 Hz, 12H). ^{13}C NMR (151 MHz, DMSO-d_6) δ 171.15, 167.94, 167.45, 165.13, 164.23, 152.23, 152.16, 132.55, 132.02, 123.15, 123.08, 123.02, 122.14, 121.65, 121.60, 113.44, 113.39, 67.48, 67.42, 42.51, 37.19, 37.00, 35.49, 34.87, 26.15, 24.81, 24.73, 23.77, 22.43, 22.38. HRMS(ESI): calcd for $C_{32}H_{45}N_5O_7$: 612.3392 (M + H$^+$), found: 612.3390 (M + H$^+$).

Compound **2**. White solid (519 mg, 66%). ^{1}H NMR (400 MHz, $CDCl_3$) δ 9.71 (s, 1H), 9.43 (s, 1H), 8.50 (d, *J* = 8.4 Hz, 1H), 8.41 – 8.34 (m, 1H), 8.26 – 8.22 (m, 1H), 8.19 – 8.15 (m, 1H), 8.13 – 8.10 (m, 1H), 8.04 (dd, *J* = 9.0, 2.7 Hz, 1H), 7.34 – 7.28 (m, 3H), 7.23 – 7.19 (m, 2H), 6.93 (d, *J* = 9.1 Hz, 1H), 6.83 (d, *J* = 9.0 Hz, 1H), 6.40 (t, *J* = 5.5 Hz, 1H), 4.90 – 4.78 (m, 1H), 4.30 (dd, *J* = 17.4, 7.3 Hz, 1H), 4.19 – 4.11 (m, 2H), 4.04 (q, *J* = 5.9 Hz, 2H), 3.93 (dd, *J* = 17.3, 4.5 Hz, 1H), 3.06 – 2.89 (m, 4H), 2.93 – 2.86 (m, 1H), 2.65 (dd, *J* = 13.7, 2.6 Hz, 1H), 2.39 (dd, *J* = 13.7, 10.6 Hz, 1H), 2.19 (s, 3H), 1.90 – 1.73 (m, 4H), 1.63 (q, *J* = 6.8 Hz, 2H), 1.09 – 0.92 (m, 12H). ^{13}C NMR (101 MHz, $CDCl_3$) δ 171.45, 169.20, 167.89, 166.39, 166.19, 153.65, 152.81, 137.14, 133.50, 129.35, 128.92, 127.18, 124.79, 124.49, 122.67, 121.09, 120.72, 112.92, 112.72, 77.36, 68.05, 67.97, 49.30, 44.49, 43.80, 42.20, 38.09, 37.85, 26.84, 25.58, 25.33, 24.56, 22.71. HRMS(ESI): calcd for $C_{39}H_{51}N_5O_7$: 702.3861 (M + H$^+$), found: 702.3871 (M + H$^+$).

Compound **15a**. To a solution of compound **10** (5.00 g, 19.76 mmol) in 60 mL dichloromethane, HBTU (8.99 g, 23.71 mmol), HOBt (3.20 g, 23.71 mmol) and *N, N*-diisopropyl-ethylamin (6.38 g,

49.40 mmol) was added at 0 °C. 30 min later, the compound **3a** (2.75 g, 19.76 mmol) in 15 mL dichloromethane was added to the mixture. Then the reaction was allowed to proceed for 6 h at room temperature and inert atmosphere. The mixture was washed with diluted HCl and saturated brine, and solvent was removed in vacuum. The crude product was purified by column chromatography (petroleum ether/acetone = 5/1) to afford a white solid (5.21 g, 78%). ^{1}H NMR (600 MHz, $CDCl_3$) δ 9.08 (d, *J* = 3.3 Hz, 1H), 8.30 (dd, *J* = 9.2, 3.2 Hz, 1H), 8.28 – 8.22 (m, 1H), 7.06 (d, *J* = 9.2 Hz, 1H), 4.27 (t, *J* = 6.7 Hz, 2H), 3.76 (q, *J* = 5.8 Hz, 2H), 3.71 (s, 3H), 2.66 (t, *J* = 5.8 Hz, 2H), 1.68 – 1.54 (m, 3H), 1.03 (d, *J* = 5.7 Hz, 6H). ^{13}C NMR (151 MHz, $CDCl_3$) δ 173.08, 163.24, 161.46, 144.80, 141.92, 128.71, 128.10, 112.60, 69.14, 51.88, 37.48, 35.42, 33.91, 25.42, 22.62. ESI-MS: calcd for $C_{16}H_{22}N_2O_6$ (M + Na$^+$) 361.14 found 361.32.

Compound **15b**. To a solution of compound **10** (5.00 g, 19.76 mmol) in 60 mL dichloromethane, HBTU (8.99 g, 23.71 mmol), HOBt (3.20 g, 23.71 mmol) and *N, N*-diisopropyl-ethylamin (6.38 g, 49.40 mmol) was added at 0 °C. 30 min later, the compound **3b** (4.53 g, 19.76 mmol) in 15 mL dichloromethane was added to the mixture. Then the reaction was allowed to proceed for 6 h at room temperature and inert atmosphere. The mixture was washed with diluted HCl and saturated brine. The combined organic layers were dried with sodium sulfate. The crude product was purified by column chromatography (petroleum ether/acetone = 5/1) to afford a white solid (5.58 g, 66%).^{1}H NMR (400 MHz, DMSO-d_6) δ 8.41 (d, *J* = 3.0 Hz, 1H), 8.34 – 8.26 (m, 2H), 7.38 (d, *J* = 9.2 Hz, 1H), 7.33 – 7.26 (m, 2H), 7.26 – 7.18 (m, 3H), 4.59 – 4.48 (m, 1H), 4.24 (t, *J* = 6.5 Hz, 2H), 3.17 (s, 3H), 2.89 (d, *J* = 6.7 Hz, 2H), 2.49 – 2.38 (m, 2H), 1.84 – 1.47 (m, 3H), 0.90 (t, *J* = 6.3 Hz, 6H).^{13}C NMR (151 MHz, DMSO-*d6*) δ 172.37, 140.39, 138.09, 129.14, 128.27, 127.57, 126.35, 125.78, 123.94, 113.80, 68.34, 48.58, 47.65, 37.90, 36.67, 24.40, 22.28, 22.19. ESI-MS: calcd for $C_{23}H_{28}N_2O_6$ (M + Na$^+$) 451.18 found 451.64.

Compound **16a**. The compound **15a** (2.0 g, 5.9 mmol) was dissolved in MeOH (100 mL), to which 2 N NaOH (11.8 mL, 23.6 mmol) was added. The mixture was heated under reflux for 2 h. After removed the MeOH under vacuum, the aqueous layer was acidulated by addition of 3 N HCl to pH 2 followed by filtration to obtain a white solid (1.5 g, 76%). ^{1}H NMR (400 MHz, DMSO-d_6) δ 12.29 (s, 1H), 8.51 (d, *J* = 3.0 Hz, 1H), 8.43 – 8.35 (m, 1H), 8.33 (dd, *J* = 9.2, 3.0 Hz, 1H), 7.40 (d, *J* = 9.3 Hz, 1H), 4.27 (t, *J* = 6.4 Hz, 2H), 3.50 (q, *J* = 6.4 Hz, 2H), 2.53 – 2.51 (m, 2H), 1.86 – 1.65 (m, 3H), 0.94 (d, *J* = 6.3 Hz, 6H). ^{13}C NMR NMR (101 MHz, DMSO-d_6) δ 173.06, 162.95, 161.21, 140.42, 127.63, 125.90, 123.77, 113.82, 113.12, 68.47, 57.06, 36.68, 35.37, 35.23, 33.61, 24.65, 22.32. HRMS(ESI): calcd for $C_{15}H_{20}N_2O_6$: 325.1394 (M + H$^+$), found: 325.1390 (M + H$^+$).

Compound **16b**. The compound **15b** (2.0 g, 4.7 mmol) was dissolved in MeOH (100 mL), to which 2 N NaOH (9.4 mL, 18.8 mmol) was added. The mixture was heated under reflux for 2 h. After removed the MeOH under vacuum, the aqueous layer was acidulated by addition of 3 N HCl to pH 2 followed by filtration to obtain a white solid. The solid was washed by methanol to get a white solid (1.4 g, 66%). ^{1}H NMR (600 MHz, DMSO-d_6) δ 12.35 (s, 1H), 8.41 (d, *J* = 3.0 Hz, 1H), 8.32 (dd, *J* = 9.2, 3.0 Hz, 1H), 8.30 (d, *J* = 8.8 Hz, 1H), 7.38 (d, *J* = 9.3 Hz, 1H), 7.34 - 7.27 (m, 2H), 7.25 – 7.18 (m, 3H), 4.57 – 4.49 (m, 1H), 4.24 (t, *J* = 6.5 Hz, 2H), 2.89 (d, *J* = 6.7 Hz, 2H), 2.54 – 2.51 (m, 1H), 2.47 – 2.42 (m, 1H), 1.75 - 1.50 (m, 3H), 0.91 (d, *J* = 6.5 Hz, 3H), 0.89 (d, *J* = 6.5 Hz, 3H). ^{13}C NMR (151 MHz, DMSO-d_6) δ 172.40, 162.46, 161.14, 140.38, 138.11, 129.16, 128.28, 127.59, 126.35, 125.79, 123.94, 113.80, 68.33, 47.65, 37.91, 36.67, 24.41, 22.29, 22.21. HRMS(ESI): calcd for $C_{22}H_{26}N_2O_6$: 413.1718 (M - H$^+$), found: 413.1720 (M - H$^+$).

Compound **17**. To a solution of Boc-glycine (13.00 g, 74.13 mmol) in 20 mL dichloromethane, HBTU (33.74 g, 88.96 mmol), HOBt (12.02 g, 88.96 mmol) and *N, N*-diisopropyl-ethylamin (23.00 g, 177.92 mmol) was added at 0 °C. 30 min later, the compound **5** (17.58 g, 74.13 mmol) in 15 mL dichloromethane was added to the mixture. Then the reaction was allowed to proceed for 8 h at room temperature and inert atmosphere. The mixture was washed with diluted HCl and saturated brine, and solvent was removed in vacuum. Purification was accomplished by chromatography on silica gel using dichloromethane/MeOH to afford **17** as a white solid (19.87 g, 68%). ^{1}H NMR (400 MHz, $CDCl_3$) δ 8.06 (s, 1H), 7.77 – 7.71 (m, 2H), 6.93 (d, *J* = 9.4 Hz, 1H), 5.22 (t, *J* = 5.6 Hz, 1H), 4.03 (t, *J* = 6.7 Hz, 2H), 3.91 (d, *J* = 5.8 Hz, 2H), 3.86 (s, 3H), 1.90 – 1.66 (m, 3H), 1.47 (s, 9H), 0.95 (d, *J* = 6.6 Hz, 6H). ^{13}C NMR (101 MHz, $CDCl_3$) δ 172.50, 167.93, 166.52, 155.82, 130.06, 125.85, 123.69, 120.68, 114.15, 68.02, 52.19, 38.00, 28.44, 25.18, 22.71. ESI-MS: calcd for $C_{20}H_{30}N_2O_6$ (M + H$^+$) 395.22 found 395.15.

Compound **18**. The compound **17** (10.00 g, 25.37 mmol) was treated with hydrochloric solution of ethyl acetic (2 N, 30 mL), then the mixture was stirred at room temperature for 1 h. Removed the solvent to obtain compound **18** as a white solid (5.95 g, 71%). ^{1}H NMR (400 MHz, DMSO-*d*$_6$) δ 10.66 (s, 1H), 8.20 (s, 3H), 7.91 (d, *J* = 2.6 Hz, 1H), 7.71 (dd, *J* = 9.0, 2.7 Hz, 1H), 7.16 (d, *J* = 9.1 Hz, 1H), 4.02 (t, *J* = 6.5 Hz, 2H), 3.82 – 3.71 (m, 5H), 1.90 – 1.73 (m, 1H), 1.65 – 1.51 (m, 2H), 0.92 (d, *J* = 6.6 Hz, 6H). ^{13}C NMR (151 MHz, DMSO-*d*$_6$) δ 165.97, 164.49, 153.95, 131.00, 124.32, 121.52, 120.22, 114.33, 67.11, 51.86, 40.82, 37.39, 24.48, 22.38. HRMS(ESI): calcd for $C_{15}H_{22}N_2O_4$: 295.1652 (M + H$^+$), found: 295.1649 (M + H$^+$).

Compound **19a**. To a solution of compound **16a** (2.00 g, 6.17 mmol) in 20 mL dichloromethane, HBTU (2.81 g, 7.40 mmol), HOBt (1.00 g, 7.40 mmol) and *N, N*-diisopropyl-ethylamin (1.99 g, 15.43 mmol) was added at 0 °C. 30 min later, the compound **18** (2.04 g, 6.17 mmol) in 15 mL dichloromethane was added to the mixture. Then the reaction was allowed to proceed for 3 h at room temperature and inert atmosphere. The mixture was washed with diluted HCl and saturated brine, and solvent was removed in vacuum. The crude product was purified by column chromatography (petroleum ether/acetone = 5/1) to afford a white solid (2.89 g, 78%). ^{1}H NMR (600 MHz, $CDCl_3$) δ 8.99 (d, *J* = 3.0 Hz, 1H), 8.30 (t, *J* = 6.0 Hz, 1H), 8.25 (dd, *J* = 9.1, 3.0 Hz, 1H), 8.21 (s, 1H), 7.72 (d, *J* = 2.8 Hz, 1H), 7.69 (dd, *J* = 8.9, 2.8 Hz, 1H), 6.99 (d, *J* = 9.1 Hz, 1H), 6.89 (d, *J* = 8.9 Hz, 1H), 6.71 (t, *J* = 5.3 Hz, 1H), 4.20 (t, *J* = 6.5 Hz, 2H), 4.08 (d, *J* = 5.6 Hz, 2H), 4.02 (t, *J* = 6.6 Hz, 2H), 3.84 (s, 3H), 3.81 (q, *J* = 6.1 Hz, 2H), 2.67 (t, *J* = 6.0 Hz, 2H), 1.90 – 1.76 (m, 4H), 1.71 (q, *J* = 6.7 Hz, 2H), 1.00 (d, *J* = 6.4 Hz, 6H), 0.96 (d, *J* = 6.6 Hz, 6H). ^{13}C NMR (151 MHz, $CDCl_3$) δ 167.54, 166.64, 161.54, 155.68, 141.57, 130.17, 128.29, 127.40, 126.87, 125.80, 123.65, 121.78, 120.45, 116.46, 113.93, 112.71, 111.91, 69.28, 67.95, 52.18, 44.39, 37.97, 37.36, 36.30, 35.95, 25.45, 25.17, 22.70, 22.62. HRMS(ESI): calcd for $C_{30}H_{40}N_4O_9$: 601.2868 (M + H$^+$), found: 601.2871 (M + H$^+$).

Compound **19b**. To a solution of compound **16b** (2.00 g, 4.83 mmol) in 20 mL dichloromethane, HBTU (2.20 g, 5.80 mmol), HOBt (0.78 g, 5.80 mmol) and *N, N*-diisopropyl-ethylamin (1.56 g, 12.08 mmol) was added at 0 °C. 30 min later, the compound **18** (1.42 g, 4.83 mmol) in 15 mL dichloromethane was added to the mixture. Then the reaction was allowed to proceed for 3 h at room temperature and inert atmosphere. The mixture was washed with diluted HCl and saturated brine, and solvent was removed in vacuum. The crude product was purified by column chromatography (petroleum ether/acetone = 5/1) to afford a white solid (2.20 g, 66%). ^{1}H NMR (600 MHz, DMSO-*d*$_6$) δ 9.70 (s, 1H), 9.26 (d, *J* = 3.0 Hz, 1H), 9.04 (d, *J* = 8.4 Hz, 1H), 8.73 (dd, *J* = 9.2, 3.0 Hz, 1H), 8.36 (d, *J* = 2.8 Hz, 1H), 8.17 (td, *J* = 8.9, 8.4, 4.0 Hz, 2H), 7.85 – 7.69 (m, 5H), 7.66 (t, *J* = 7.3 Hz, 1H), 7.47 (d, *J* = 9.0 Hz, 1H), 5.27 – 5.13 (m, 1H), 4.86 – 4.69 (m, 2H), 4.59 – 4.42 (m, 4H), 4.24 (s,

3H), 3.52 (d, J = 7.0 Hz, 2H), 3.11 (dd, J = 15.0, 5.2 Hz, 1H), 3.03 (dd, J = 15.0, 6.1 Hz, 1H), 2.41 – 2.17 (m, 4H), 2.11 (q, J = 6.6 Hz, 2H), 1.42 – 1.38 (m, 12H) ^{13}C NMR (151 MHz, DMSO-d_6) δ 181.84, 177.97, 176.74, 172.84, 172.28, 165.05, 151.75, 149.18, 142.18, 139.98, 138.90, 138.18, 137.76, 136.89, 135.05, 133.48, 132.79, 131.46, 124.45, 124.04, 79.39, 77.94, 61.69, 59.48, 53.75, 50.28, 49.01, 48.43, 47.49, 35.38, 35.33, 32.50, 32.30. HRMS(ESI): calcd for $C_{37}H_{46}N_4O_9$: 691.3338 (M + H$^+$), found: 691.3338 (M + H$^+$).

Compound **20a**. The ester **19a** (2.0 g, 3.3 mmol) was dissolved in MeOH (100 mL), to which 2 N NaOH (6.7 mL, 13.3 mmol) was added. The mixture was heated under reflux for 2 h. After removed the MeOH under vacuum, the aqueous layer was acidulated by addition of 3 N HCl to pH 2 followed by filtration to obtain a white solid (1.5 g, 79%). ^{1}H NMR (600 MHz, DMSO-d_6) δ 9.99 – 9.87 (m, 1H), 8.56 (d, J = 3.1 Hz, 1H), 8.41 (t, J = 5.8 Hz, 1H), 8.37 – 8.24 (m, 2H), 7.84 (t, J = 2.9 Hz, 1H), 7.65 (dt, J = 8.9, 2.2 Hz, 1H), 7.38 (d, J = 9.2 Hz, 1H), 7.06 (d, J = 9.0 Hz, 1H), 4.32 – 4.20 (m, 2H), 4.04 – 3.98 (m, 4H), 3.87 (dd, J = 6.0, 3.1 Hz, 2H), 3.56 – 3.47 (m, 2H), 1.87 – 1.76 (m, 1H), 1.76 – 1.66 (m, 3H), 1.63 – 1.53 (m, 2H), 0.96 – 0.88 (m, 12H). ^{13}C NMR NMR (151 MHz, DMSO-d_6) δ 171.13, 167.49, 167.13, 162.81, 161.26, 153.37, 140.41, 131.51, 127.64, 126.07, 126.02, 123.72, 123.62, 121.57, 114.04, 113.80, 113.10, 68.51, 67.10, 57.03, 42.54, 37.47, 36.60, 35.95, 35.76, 34.75, 34.69, 34.50, 24.67, 24.43, 22.42, 22.32. HRMS(ESI): calcd for $C_{29}H_{38}N_4O_9$: 587.2712 (M + H$^+$), found: 587.2716 (M + H$^+$).

Compound **20b**. The ester **19b** (2.0 g, 2.9 mmol) was dissolved in MeOH (100 mL), to which 2 N NaOH (5.8 mL, 11.6 mmol) was added. The mixture was heated under reflux for 2 h. After removed the MeOH under vacuum, the aqueous layer was acidulated by addition of 3 N HCl to pH 2 followed by filtration to obtain a white solid (1.5 g, 78%). ^{1}H NMR (400 MHz, DMSO-d_6) δ 9.92 (s, 1H), 8.57 – 8.40 (m, 3H), 8.29 (td, J = 9.2, 8.4, 3.0 Hz, 1H), 7.67 (s, 1H), 7.52 (dd, J = 8.9, 2.7 Hz, 1H), 7.41 – 7.14 (m, 6H), 6.91 (d, J = 8.9 Hz, 1H), 4.61 – 4.44 (m, 1H), 4.29 – 4.14 (m, 2H), 3.96 (t, J = 6.7 Hz, 2H), 3.87 (d, J = 5.7 Hz, 2H), 2.96 – 2.82 (m, 2H), 2.48 – 2.36 (m, 2H), 1.86 – 1.73 (m, 1H), 1.73 – 1.59 (m, 3H), 1.59 – 1.51 (m, 2H), 0.93 – 0.83 (m, 12H). ^{13}C NMR NMR (101 MHz, DMSO-d_6) δ 181.84, 178.07, 172.82, 172.29, 164.59, 151.77, 149.18, 143.05, 140.00, 138.91, 138.17, 137.80, 136.90, 135.75, 133.76, 133.50, 132.64, 124.40, 124.06, 79.42, 78.94, 59.46, 53.79, 50.35, 49.06, 48.19, 47.53, 35.46, 35.41, 32.53, 32.49, 32.34. HRMS(ESI): calcd for $C_{36}H_{44}N_4O_9$: 677.3181 (M + H$^+$), found: 677.3174 (M + H$^+$).

General procedure for the synthesis of compounds 21. To a solution of compound **20** (1.12 mmol) in 30 mL dichloromethane, HBTU (511 mg, 1.35 mmol), HOBt (182 mg, 1.35 mmol) and *N, N*-diisopropyl- ethylamine (363 mg, 2.81 mmol) was added at 0 °C. 30 min later, (*S*)-2-amino-*N*,4-dimethylpentanamide (1.12 mmol) in 15 mL dichloromethane was added to the mixture. Then the reaction was allowed to proceed for 3 h at room temperature and inert atmosphere. The mixture was washed with diluted HCl and saturated brine, and solvent was removed in vacuum. The crude product was purified by column chromatography (petroleum ether/ acetone = 3/1).

Compound **21a**. White solid (479 mg, 60%). ^{1}H NMR (400 MHz, $CDCl_3$) δ 9.12 – 8.98 (m, 1H), 8.95 (s, 1H), 8.57 (d, J = 7.5 Hz, 1H), 8.33 – 8.25 (m, 1H), 8.22 (d, J = 8.6 Hz, 1H), 7.99 (d, J = 8.6 Hz, 1H), 7.91 (s, 1H), 7.29 (d, J = 3.3 Hz, 1H), 6.99 (d, J = 8.5 Hz, 2H), 6.92 (d, J = 8.6 Hz, 1H), 4.74 – 4.60 (m, 1H), 4.25 – 4.05 (m, 6H), 3.86 – 3.74 (m, 2H), 2.92 – 2.75 (m, 3H), 2.69 (s, 2H), 1.88 – 1.77 (m, 9H), 1.01 – 0.86 (m, 18H). ^{13}C NMR (101 MHz, $CDCl_3$) δ 172.66, 172.00, 167.49, 165.07, 163.76, 161.49, 141.83, 131.62, 128.41, 128.08, 125.70, 123.50, 122.55, 120.86, 113.10, 112.73,

69.22, 68.26, 52.62, 44.24, 41.88, 37.99, 37.52, 26.37, 25.49, 25.35, 25.03, 23.09, 22.70, 22.63, 22.57, 22.28. HRMS(ESI): calcd for $C_{36}H_{52}N_6O_9$: 713.3869 ($M + H^+$), found: 713.3873 ($M + H^+$).

Compound **21b**. White solid (683 mg, 76%). ^{1}H NMR (600 MHz, $CDCl_3$) δ 8.98 – 8.83 (m, 1H), 8.76 – 8.63 (m, 1H), 8.60 – 8.30 (m, 1H), 8.29 – 8.22 (m, 1H), 8.18 (d, J = 9.1 Hz, 1H), 8.00 – 7.87 (m, 2H), 7.23 (s, 5H), 6.96 (d, J = 9.1 Hz, 1H), 6.89 (s, 1H), 6.79 (s, 1H), 4.89 – 4.75 (m, 1H), 4.75 – 4.58 (m, 1H), 4.28 – 4.07 (m, 6H), 4.07 – 3.84 (m, 1H), 3.17 – 2.89 (m, 2H), 2.78 (d, J = 12.4 Hz, 3H), 2.71 – 2.46 (m, 2H), 1.81 – 1.67 (m, 9H), 1.16 – 0.89 (m, 18H). ^{13}C NMR (151 MHz, $CDCl_3$) δ 172.69, 171.52, 165.12, 163.23, 161.49, 154.10, 141.72, 137.45, 131.53, 129.53, 128.87, 128.51, 128.08, 127.07, 125.95, 123.77, 122.31, 120.79, 112.97, 112.73, 69.12, 68.20, 52.49, 49.03, 44.33, 41.61, 40.57, 38.00, 37.40, 29.86, 26.41, 25.33, 25.23, 25.01, 23.16, 22.70, 22.64, 22.59, 22.20. HRMS(ESI): calcd for $C_{43}H_{58}N_6O_9$: 803.4338 ($M + H^+$), found: 803.4341 ($M + H^+$).

General procedure for the synthesis of compounds 22. Compound **21** (1.40 mmol) was reduced by catalytic hydrogenation in dichloromethane (50 mL) at room temperature and atmospheric pressure, using Pd-C (10%) as the catalyst. After all substrate was reduced, removal of the catalyst and solvent gave the product as a yellow solid. These compounds were used without further purification.

General procedure for the synthesis of compounds 1a and **2a**. To a solution of compound **22** (1.39 mmol) in 30 mL dichloromethane, HBTU (633 mg, 1.67 mmol), HOBt (226 mg, 1.67 mmol) and *N, N*-diisopropyl- ethylamine (484 mg, 3.48 mmol) was added at 0 °C. 30 min later, acetyl-*L*-valine (1.39 mmol) in 15 mL dichloromethane was added to the mixture. Then the reaction was allowed to proceed for 3 h at room temperature and inert atmosphere. The mixture was washed with diluted HCl and saturated brine, and solvent was removed in vacuum. The crude product was purified by column chromatography (petroleum ether/ acetone = 2/1).

Compound **1a**. White solid (687 mg, 60%). ^{1}H NMR (400 MHz, $CDCl_3$) δ 10.04 (s, 1H), 9.47 (s, 1H), 8.58 (s, 1H), 8.53 (d, J = 7.4 Hz, 1H), 8.20 (d, J = 7.2 Hz, 1H), 8.13 – 7.99 (m, 2H), 7.91 (d, J = 8.8 Hz, 1H), 7.70 – 7.45 (m, 1H), 7.19 – 7.06 (m, 1H), 6.98 – 6.82 (m, 2H), 6.54 (d, J = 8.8 Hz, 1H), 4.80 – 4.67 (m, 2H), 4.23 – 4.02 (m, 6H), 4.01 – 3.87 (m, 1H), 3.73 – 3.55 (m, 1H), 2.73 (d, J = 4.5 Hz, 3H), 2.71 – 2.61 (m, 1H), 2.61 – 2.51 (m, 1H), 2.11 (s, 3H), 1.95 – 1.56 (m, 10H), 1.04 – 0.90 (m, 24H). ^{13}C NMR (151 MHz, $CDCl_3$) δ 173.14, 172.45, 171.16, 170.63, 168.21, 166.05, 165.38, 154.13, 153.79, 131.95, 131.55, 126.56, 125.66, 124.17, 123.79, 121.05, 120.70, 113.02, 112.83, 68.21, 68.08, 59.02, 52.61, 44.15, 41.82, 37.96, 37.80, 37.03, 36.71, 32.11, 26.33, 25.49, 25.44, 25.25, 24.97, 23.40, 23.10, 22.70, 22.67, 22.53, 22.48, 22.19, 19.33, 18.42. HRMS(ESI): calcd for $C_{43}H_{65}N_7O_9$: 824.4917 ($M + H^+$), found: 824.4912 ($M + H^+$).

Compound **2a**. Yellow solid (965 mg, 76%). ^{1}H NMR (400 MHz, $CDCl_3$) δ 10.11 (s, 1H), 9.63 (s, 1H), 8.54 (d, J = 7.1 Hz, 1H), 8.48 (d, J = 8.0 Hz, 1H), 8.36 (d, J = 8.6 Hz, 1H), 8.22 – 8.15 (m, 1H), 8.15 – 8.08 (m, 1H), 7.99 – 7.87 (m, 1H), 7.77 – 7.65 (m, 1H), 7.25 – 7.16 (m, 5H), 6.98 (d, J = 9.0 Hz, 1H), 6.92 (d, J = 9.1 Hz, 1H), 6.49 – 6.24 (m, 2H), 4.93 – 4.66 (m, 3H), 4.30 (dd, J = 17.2, 7.5 Hz, 1H), 4.24 – 4.02 (m, 4H), 3.90 (dd, J = 17.2, 3.9 Hz, 1H), 2.98 (dd, J = 13.8, 6.5 Hz, 1H), 2.87 (dd, J = 13.8, 7.3 Hz, 1H), 2.69 (d, J = 4.5 Hz, 3H), 2.59 (d, J = 11.7 Hz, 1H), 2.38 – 2.23 (m, 1H), 2.10 (s, 3H), 1.91 – 1.62 (m, 10H), 1.02 – 0.92 (m, 24H). ^{13}C NMR (101 MHz, $CDCl_3$) δ 173.36, 171.75, 171.28, 170.73, 168.63, 165.39, 165.31, 154.24, 153.83, 137.04, 132.05, 131.35, 129.35, 128.64, 127.20, 126.88, 125.27, 124.60, 123.78, 120.62, 120.57, 113.05, 112.75, 68.08, 68.01, 59.18, 52.71, 48.93, 43.90, 42.12, 41.76, 41.22, 37.89, 37.60, 31.90, 26.28, 25.14, 24.90, 23.18, 23.06,

22.64, 22.58, 22.52, 22.46, 22.02, 19.30, 18.40. HRMS(ESI): calcd for $C_{50}H_{71}N_7O_9$: 914.5386 (M + H^+), found: 914.5380 (M + H^+).

Compound **23**. Compound **10** (5.00 g, 19.76 mmol) was reduced by catalytic hydrogenation in dichloromethane (50 mL) at room temperature and atmospheric pressure, using Pd-C (0.50 g, 10%) as the catalyst. After all substrate was reduced, removal of catalyst and solvent gave the product as yellow oil. This compound was used without further purification.

Compound **24** was synthesized according to previously reported procedures.[3]

Compound **2b**. White solid. 1H NMR (600 MHz, $CDCl_3$) δ 10.08 (s, 1H), 9.53 (s, 1H), 8.60 – 8.44 (m, 2H), 8.23 (dd, J = 9.0, 2.7 Hz, 1H), 8.14 – 8.05 (m, 2H), 7.87 (dd, J = 8.9, 2.7 Hz, 1H), 7.76 – 7.64 (m, 1H), 7.52 – 7.36 (m, 1H), 7.22 – 7.05 (m, 5H), 6.95 (d, J = 8.9 Hz, 1H), 6.91 – 6.82 (m, 2H), 4.90 – 4.79 (m, 1H), 4.79 – 4.68 (m, 1H), 4.68 – 4.57 (m, 1H), 4.25 – 3.80 (m, 6H), 2.96 – 2.80 (m, 2H), 2.70 (d, J = 4.6 Hz, 3H), 2.61 (dd, J = 14.1, 4.2 Hz, 1H), 2.37 (dd, J = 14.0, 9.1 Hz, 1H), 2.07(s, 3H), 1.86 - 1.53 (m, 10H), 0.97 – 0.88 (m, 24H). 13C NMR (151 MHz, $CDCl_3$) δ 173.36, 171.75, 171.29, 170.73, 168.64, 165.40, 165.31, 159.56, 154.24, 153.84, 137.05, 132.05, 131.35, 129.35, 128.65, 127.20, 126.88, 125.27, 124.61, 123.78, 120.63, 120.58, 113.06, 112.76, 68.08, 68.01, 59.18, 52.71, 48.93, 43.90, 42.10, 41.75, 41.21, 37.89, 37.60, 37.48, 31.89, 26.28, 25.14, 25.06, 24.90, 23.18, 23.06, 22.64, 22.57, 22.52, 22.46, 22.02, 21.86, 19.29, 19.18, 18.40. HRMS(ESI): calcd for $C_{50}H_{71}N_7O_9$: 914.5386 (M + H^+), found: 914.5380 (M + H^+).

Compound **1'**. White solid. 1H NMR (400 MHz, $CDCl_3$) δ 8.18 (s, 1H), 7.34 (d, J = 8.5 Hz, 1H), 6.88 (s, 1H), 6.47 (d, J = 6.8 Hz, 1H), 5.10 – 4.97 (m, 1H), 4.16 – 4.04 (m, 2H), 3.86 – 3.68 (m, 2H), 3.37 – 3.22 (m, 1H), 3.11 (s, 3H), 2.94 (s, 3H), 2.60 – 2.48 (m, 1H), 2.33 – 2.24 (m, 1H), 2.05 – 1.89 (m, 4H), 1.74 – 1.57 (m, 1H), 1.57 – 1.42 (m, 1H), 1.42 – 1.29 (m, 1H), 0.96 (d, J = 6.7 Hz, 9H), 0.87 (d, J = 6.7 Hz, 3H). 13C NMR (151 MHz, $CDCl_3$) δ 173.13, 172.76, 171.63, 169.44, 59.75, 47.56, 43.56, 41.66, 37.25, 36.54, 36.36, 36.05, 30.59, 24.75, 23.39, 22.80, 21.76, 19.26, 18.87. HRMS(ESI): calcd for $C_{20}H_{37}N_5O_5$: 428.2867 (M + H^+), found: 428.2870 (M + H^+).

Compound **2'**. White solid. 1H NMR (400 MHz, $CDCl_3$) δ 7.69 (d, J = 7.5 Hz, 1H), 7.24 – 7.14 (m, 6H), 6.66 (s, 1H), 6.23 (d, J = 7.3 Hz, 1H), 5.05 – 4.95 (m, 1H), 4.29 – 4.17 (m, 1H), 4.14 (t, J = 7.1 Hz, 1H), 3.99 – 3.84 (m, 2H), 3.17 – 3.05 (m, 4H), 3.00 – 2.87 (m, 4H), 2.61 (dd, J = 14.0, 7.0 Hz, 1H), 2.49 – 2.37 (m, 1H), 1.99 (s, 3H), 1.96 – 1.85 (m, 1H), 1.73 – 1.58 (m, 1H), 1.54 – 1.44 (m, 1H), 1.44 – 1.34 (m, 1H), 0.97 (d, J = 6.4 Hz, 3H), 0.89 (d, J = 6.6 Hz, 3H), 0.80 (dd, J = 11.5, 6.8 Hz, 6H). 13C NMR (151 MHz, $CDCl_3$) δ 172.81, 171.88, 171.59, 171.10, 169.07, 138.22, 129.45, 128.56, 126.65, 59.40, 49.78, 47.60, 43.54, 42.03, 39.90, 39.37, 37.27, 36.05, 30.80, 24.82, 23.45, 23.09, 21.91, 19.21, 18.40. HRMS(ESI): calcd for $C_{27}H_{43}N_5O_5$: 518.3337 (M + H^+), found: 518.3334 (M + H^+).

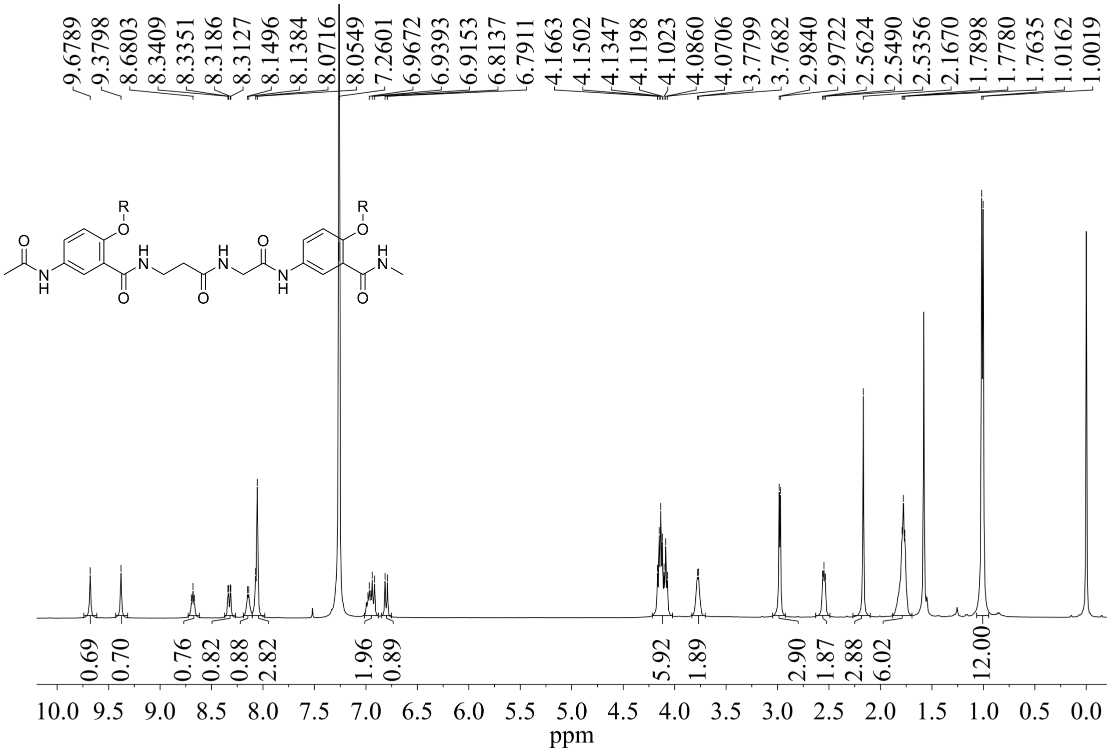

Supplementary Figure 6. ^{1}H NMR spectra of compound **1** ($CDCl_3$, 298 K, 400 MHz)

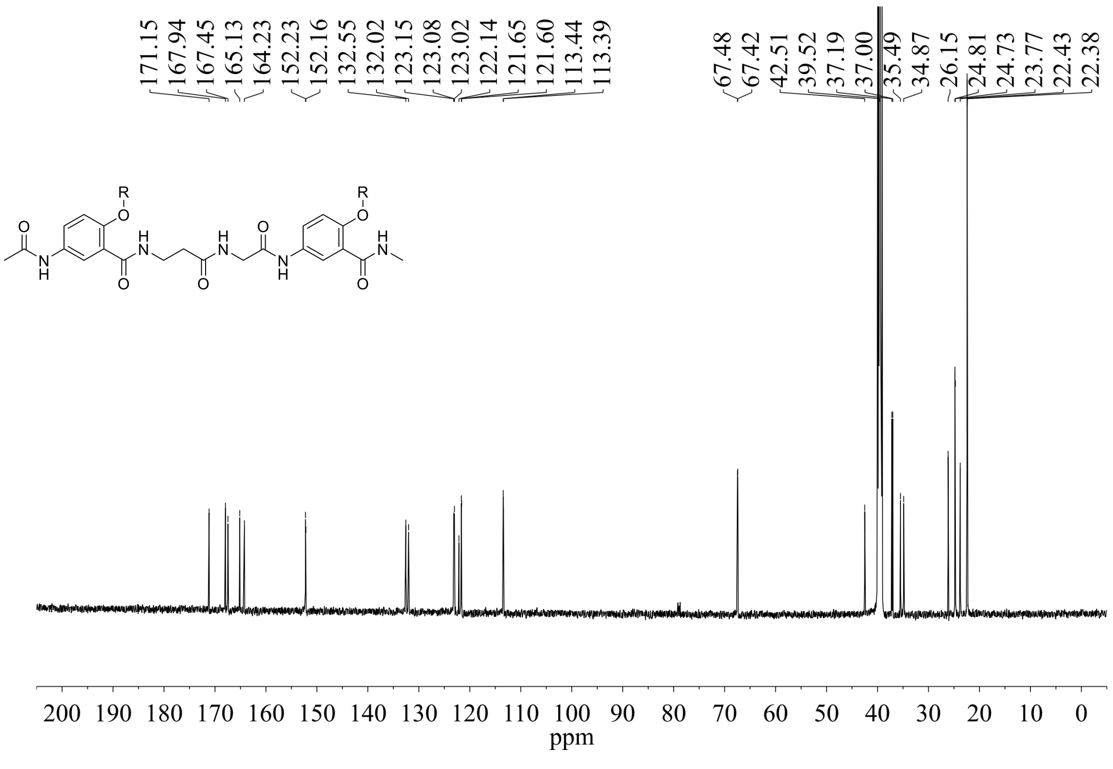

Supplementary Figure 7. ^{13}C NMR spectra of compound **1** (DMSO-d_6, 299 K, 151 MHz)

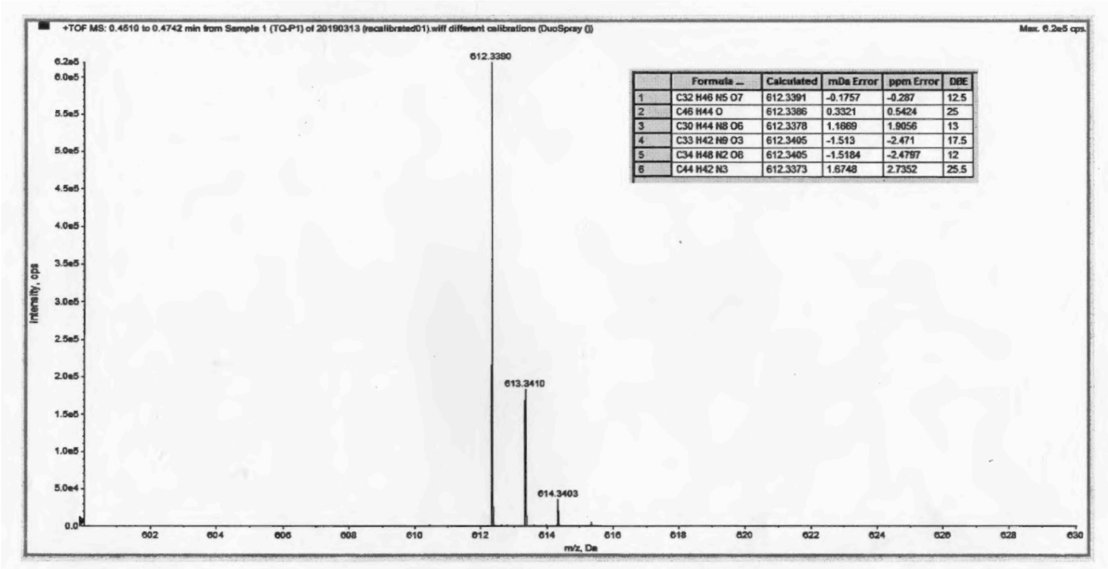

	Formula ...	Calculated	mDa Error	ppm Error	DBE
1	C32 H46 N5 O7	612.3391	-0.1757	-0.287	12.5
2	C46 H44 O	612.3386	0.3321	0.5424	25
3	C30 H44 N8 O6	612.3378	1.1669	1.9056	13
4	C33 H42 N9 O3	612.3405	-1.513	-2.471	17.5
5	C34 H48 N2 O8	612.3405	-1.5184	-2.4797	12
6	C44 H42 N3	612.3373	1.6748	2.7352	25.5

Supplementary Figure 8. MS spectra of compound **1**

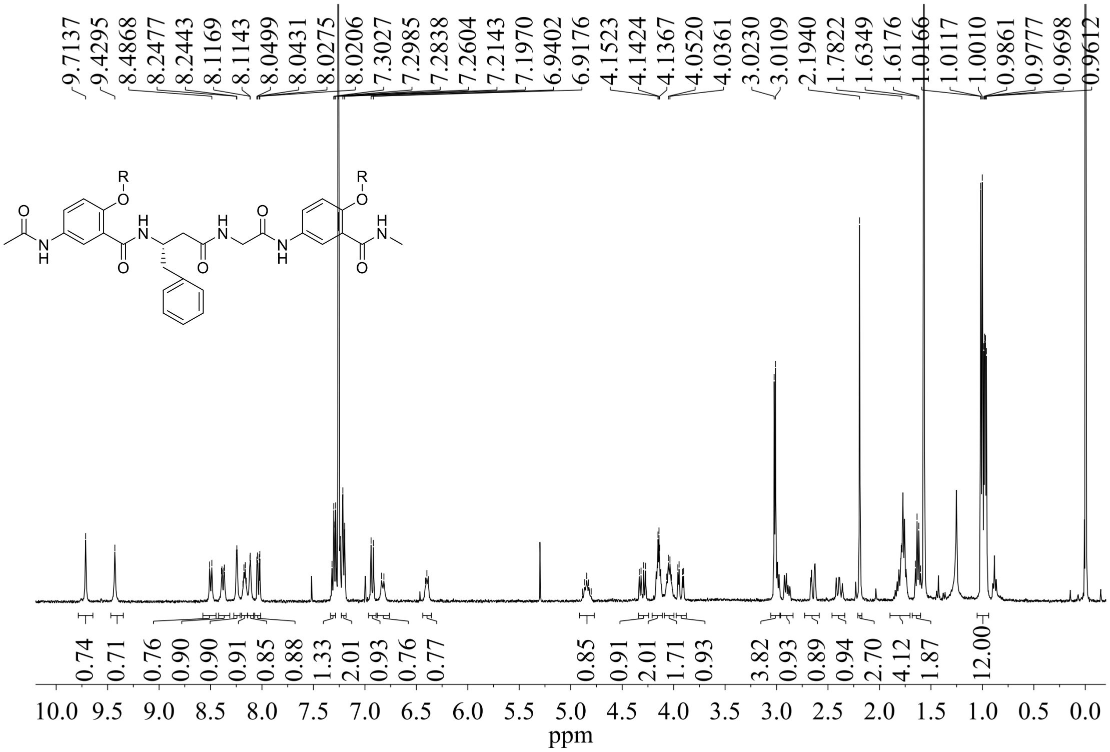

Supplementary Figure 9. ^{1}H NMR spectra of compound **2** ($CDCl_3$, 296 K, 400 MHz)

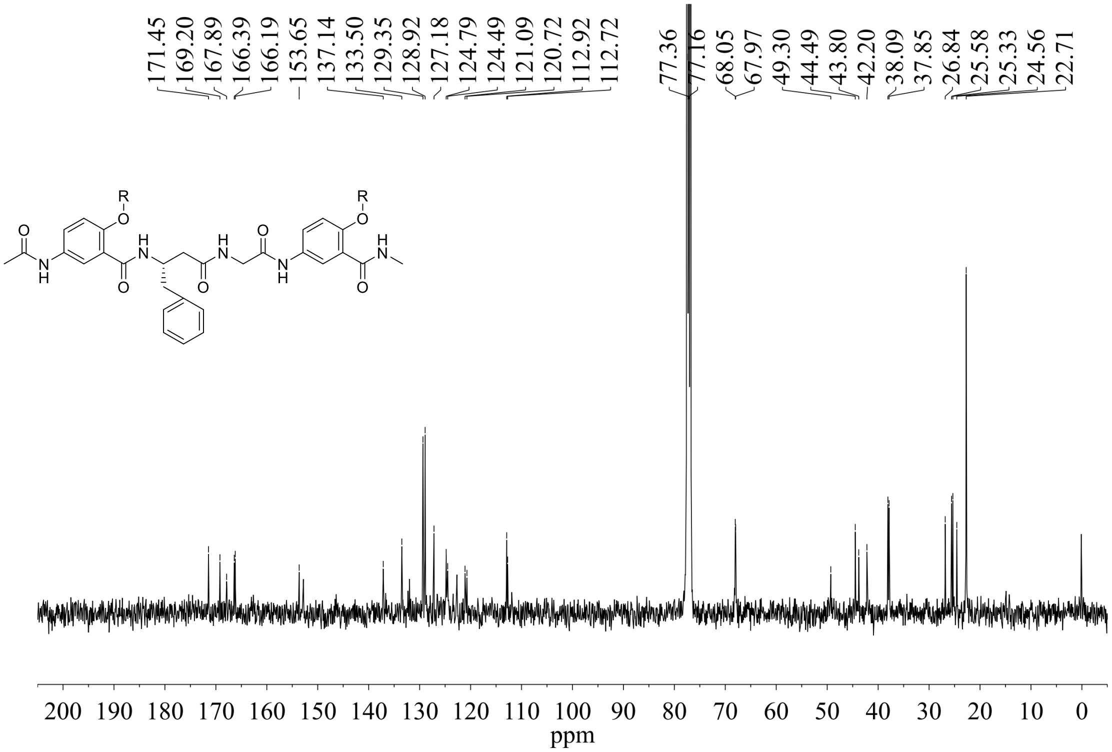

Supplementary Figure 10. ^{13}C NMR spectra of compound **2** ($CDCl_3$, 295 K, 101 MHz)

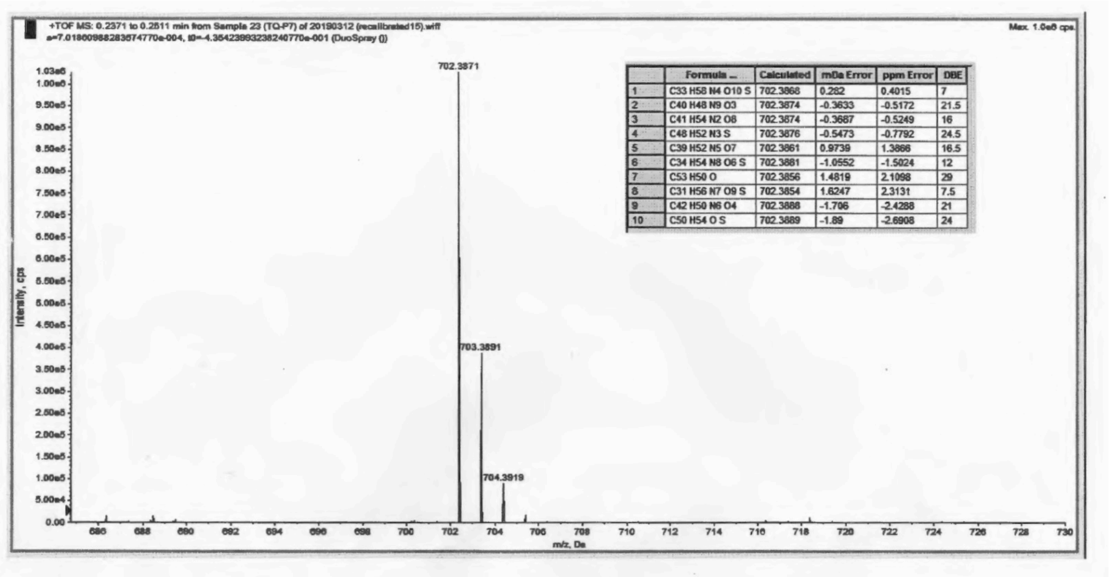

	Formula ...	Calculated	mDa Error	ppm Error	DBE
1	C33 H58 N4 O10 S	702.3868	0.282	0.4015	7
2	C40 H48 N9 O3	702.3874	-0.3633	-0.5172	21.5
3	C41 H54 N2 O8	702.3874	-0.3687	-0.5249	16
4	C48 H52 N3 S	702.3876	-0.5473	-0.7792	24.5
5	C39 H52 N5 O7	702.3861	0.9739	1.3866	16.5
6	C34 H54 N8 O6 S	702.3881	-1.0552	-1.5024	12
7	C53 H50 O	702.3856	1.4819	2.1098	29
8	C31 H56 N7 O9 S	702.3854	1.6247	2.3131	7.5
9	C42 H50 N6 O4	702.3888	-1.706	-2.4288	21
10	C50 H54 O S	702.3889	-1.89	-2.6908	24

Supplementary Figure 11. MS spectra of compound **2**

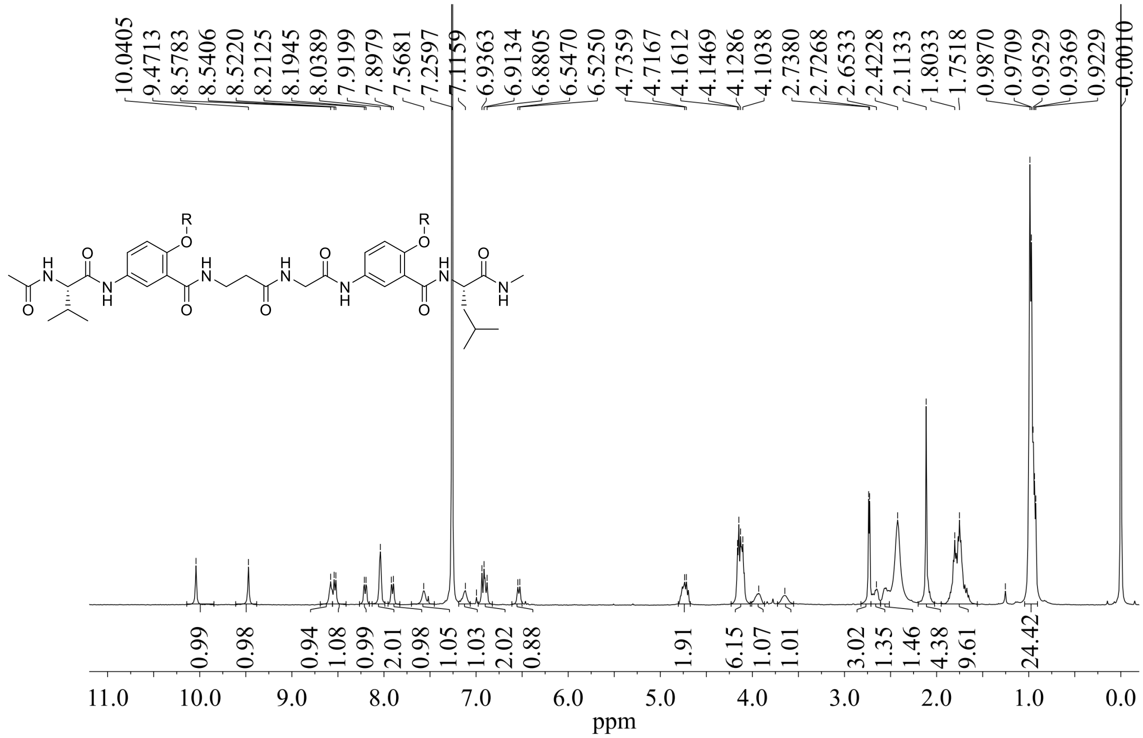

Supplementary Figure 22. ^{1}H NMR spectra of compound **1a** ($CDCl_3$, 298 K, 400 MHz)

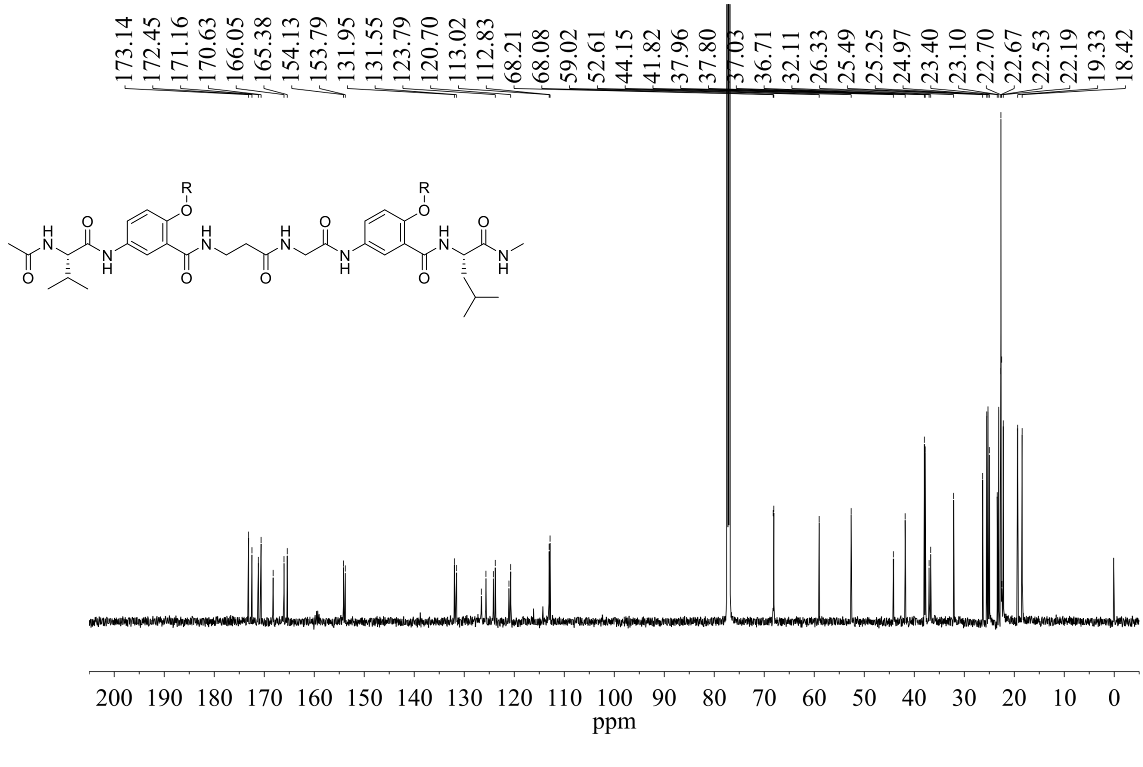

Supplementary Figure 33. ^{13}C NMR spectra of compound **1a** ($CDCl_3$, 299 K, 151 MHz)

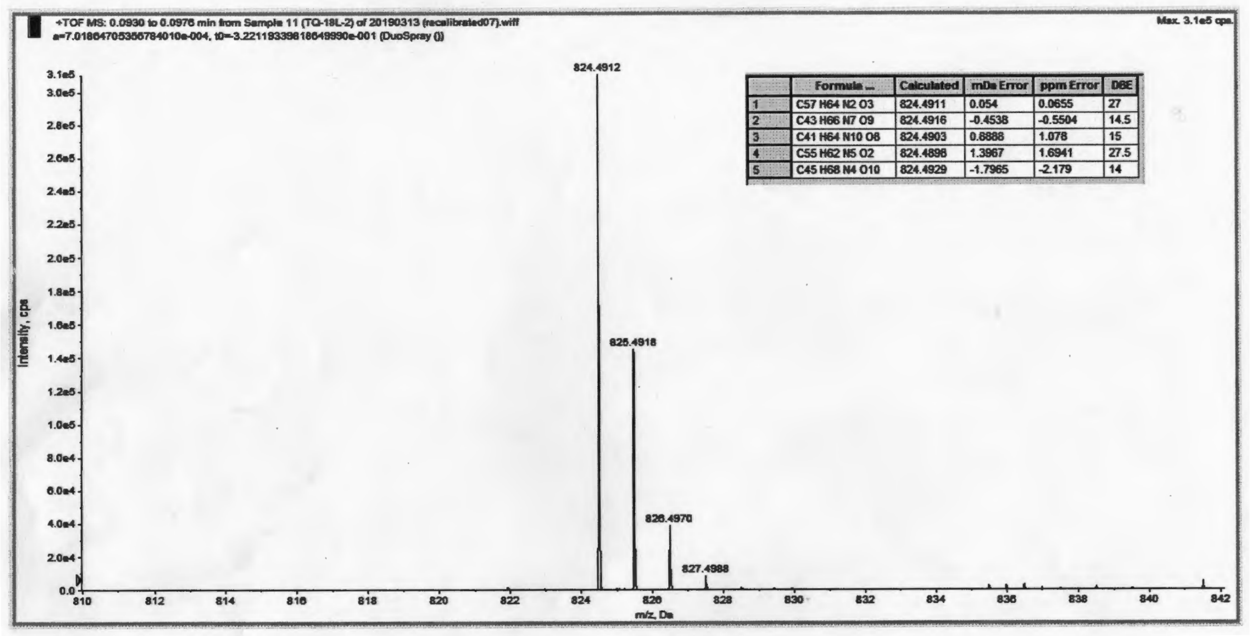

	Formula ...	Calculated	mDa Error	ppm Error	DBE
1	C57 H64 N2 O3	824.4911	0.054	0.0655	27
2	C43 H66 N7 O9	824.4916	-0.4538	-0.5504	14.5
3	C41 H64 N10 O8	824.4903	0.8888	1.078	15
4	C55 H62 N5 O2	824.4898	1.3967	1.6941	27.5
5	C45 H68 N4 O10	824.4929	-1.7965	-2.179	14

Supplementary Figure 44. MS spectra of compound **1a**

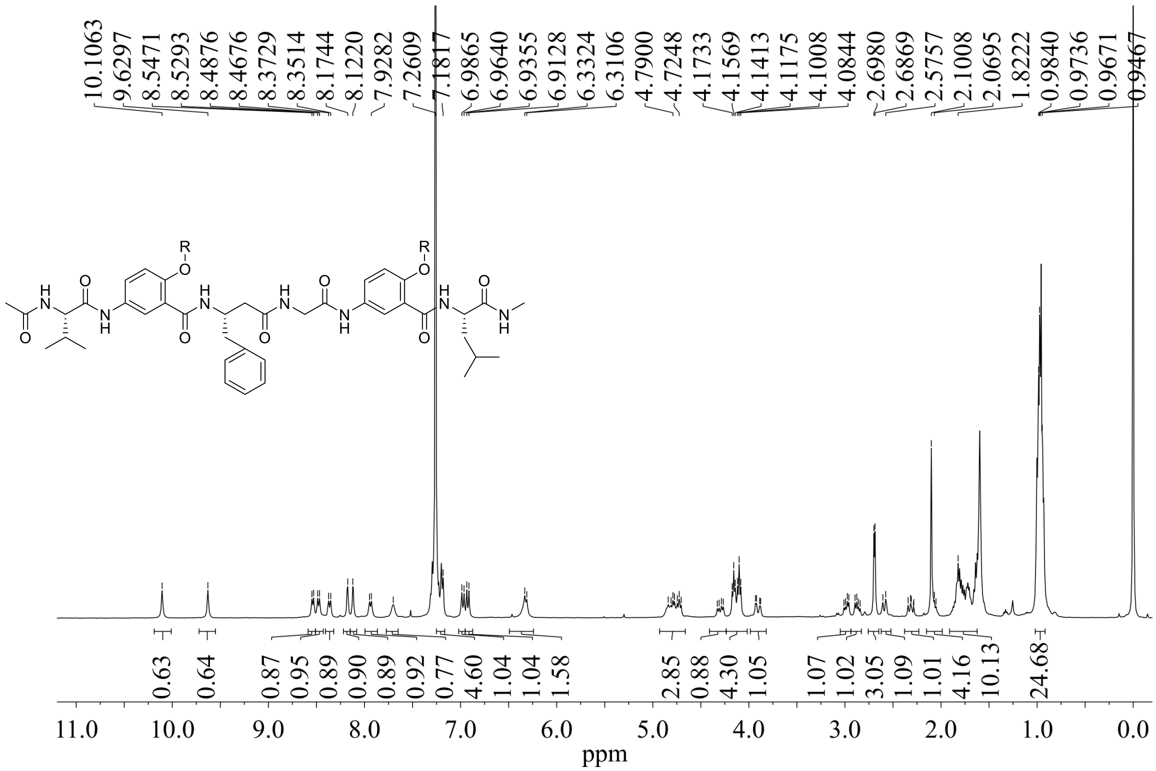

Supplementary Figure 55. ^{1}H NMR spectra of compound **2a** ($CDCl_3$, 298 K, 400 MHz)

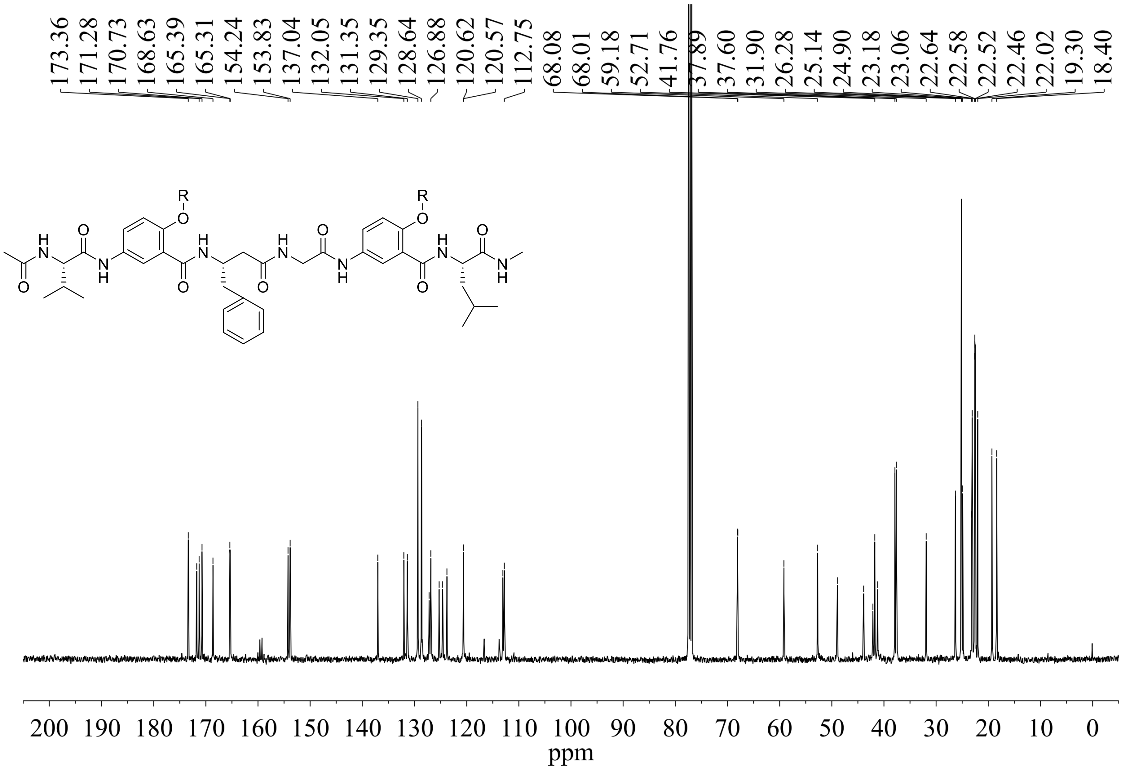

Supplementary Figure 66. ^{13}C NMR spectra of compound **2a** ($CDCl_3$, 299 K, 101 MHz)

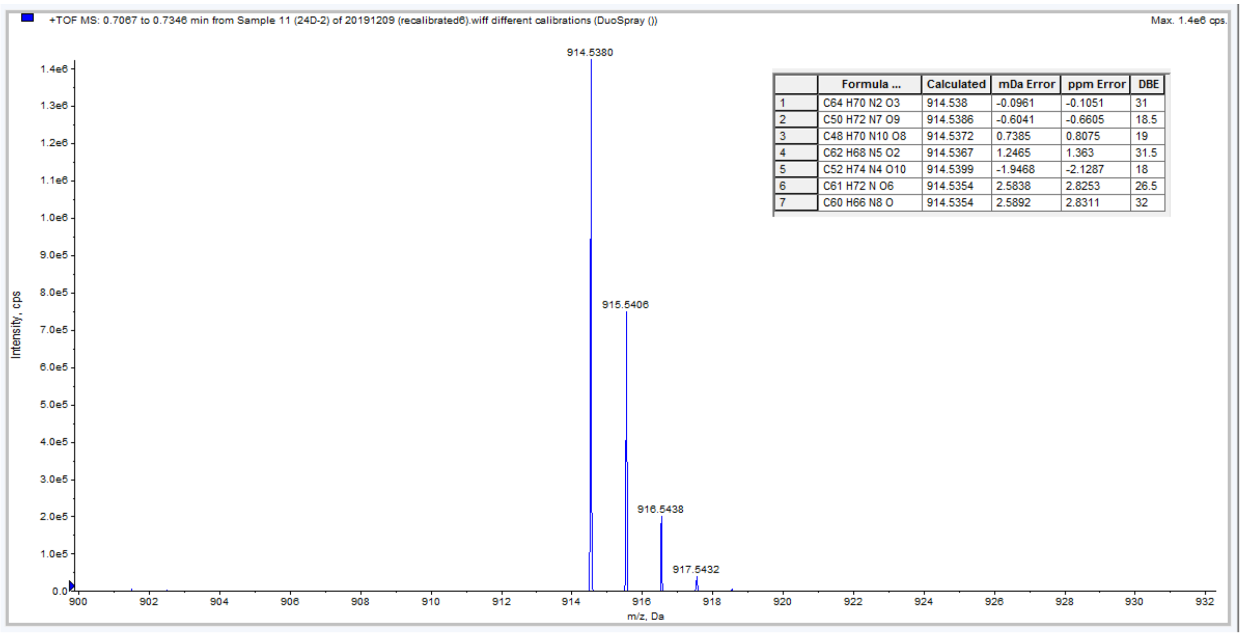

	Formula ...	Calculated	mDa Error	ppm Error	DBE
1	C64 H70 N2 O3	914.538	-0.0961	-0.1051	31
2	C50 H72 N7 O9	914.5386	-0.6041	-0.6605	18.5
3	C48 H70 N10 O8	914.5372	0.7385	0.8075	19
4	C62 H68 N5 O2	914.5367	1.2465	1.363	31.5
5	C52 H74 N4 O10	914.5399	-1.9468	-2.1287	18
6	C61 H72 N O6	914.5354	2.5838	2.8253	26.5
7	C60 H66 N8 O	914.5354	2.5892	2.8311	32

Supplementary Figure 77. MS spectra of compound **2a**

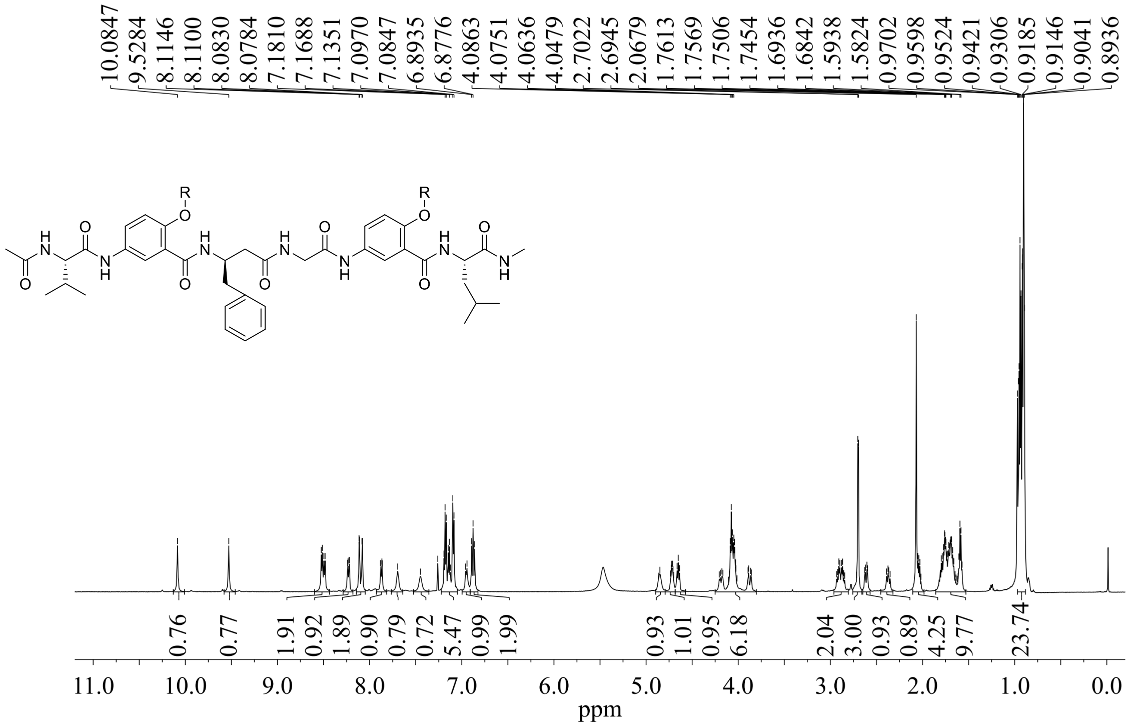

Supplementary Figure 88. ^{1}H NMR spectra of compound **2b** ($CDCl_3$, 298 K, 600 MHz)

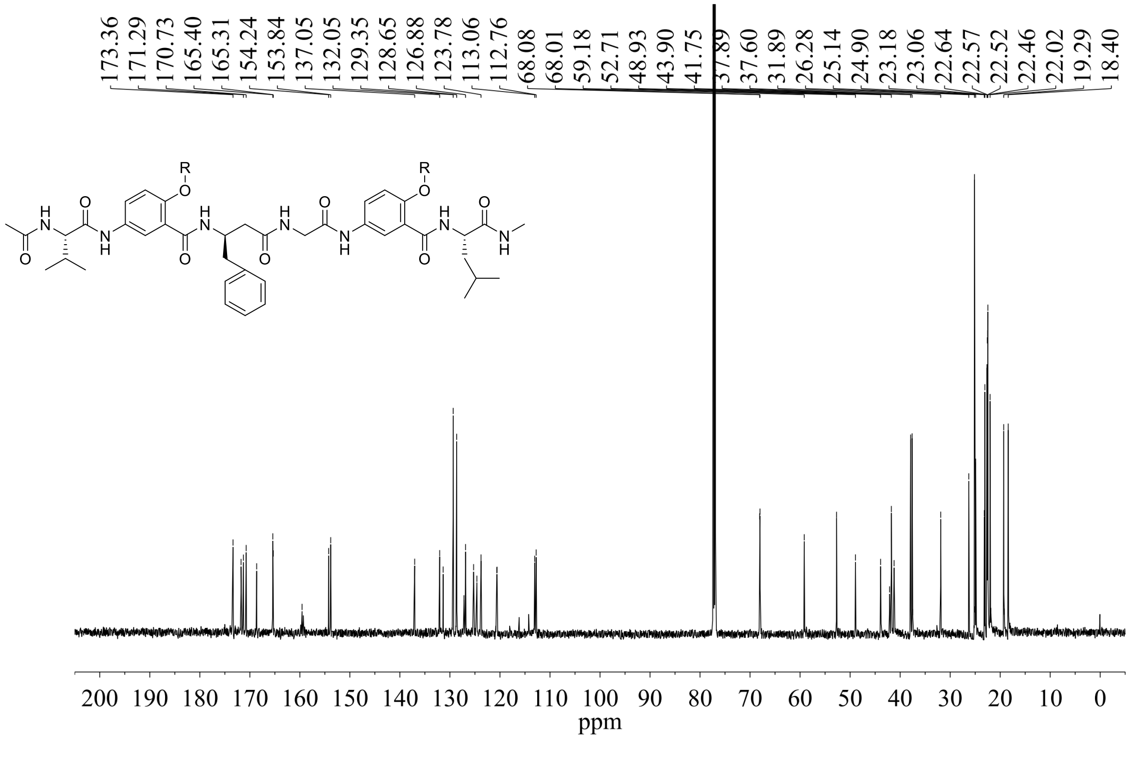

Supplementary Figure 99. ^{13}C NMR spectra of compound **2b** ($CDCl_3$, 299 K, 151 MHz)

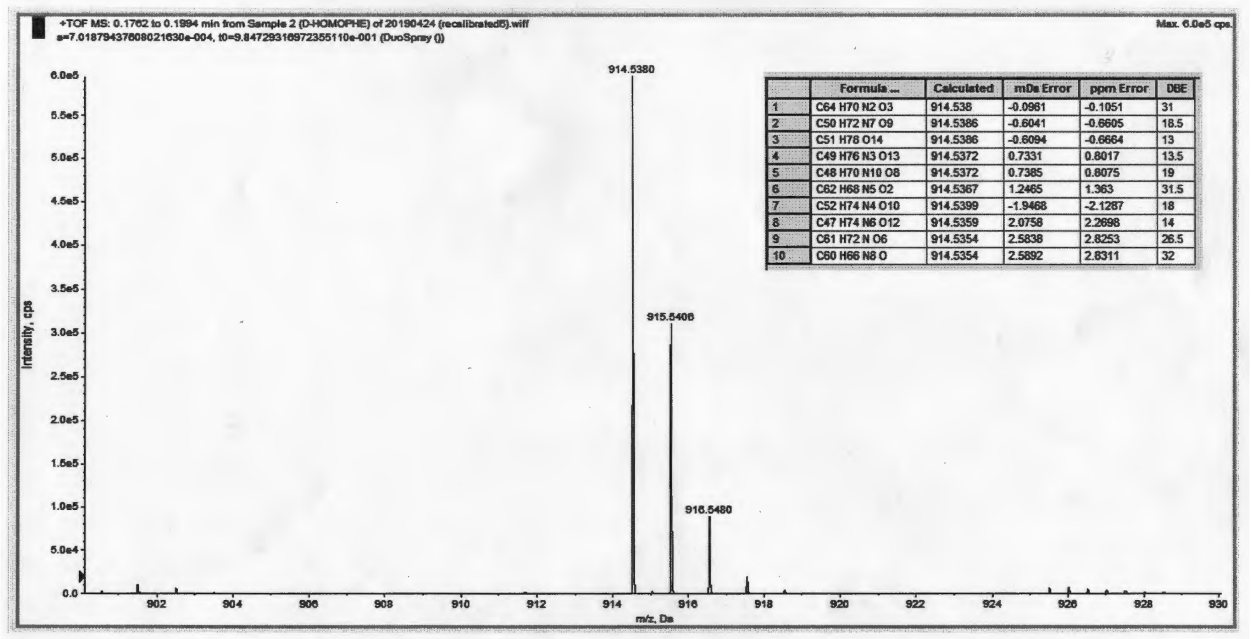

	Formula ...	Calculated	mDa Error	ppm Error	DBE
1	C64 H70 N2 O3	914.538	-0.0961	-0.1051	31
2	C50 H72 N7 O9	914.5386	-0.6041	-0.6605	18.5
3	C51 H78 O14	914.5386	-0.6094	-0.6664	13
4	C49 H76 N3 O13	914.5372	0.7331	0.8017	13.5
5	C48 H70 N10 O8	914.5372	0.7385	0.8075	19
6	C62 H68 N5 O2	914.5367	1.2465	1.363	31.5
7	C52 H74 N4 O10	914.5399	-1.9468	-2.1287	18
8	C47 H74 N6 O12	914.5359	2.0758	2.2698	14
9	C61 H72 N O6	914.5354	2.5838	2.8253	26.5
10	C60 H66 N8 O	914.5354	2.5892	2.8311	32

Supplementary Figure 20. MS spectra of compound **2b**

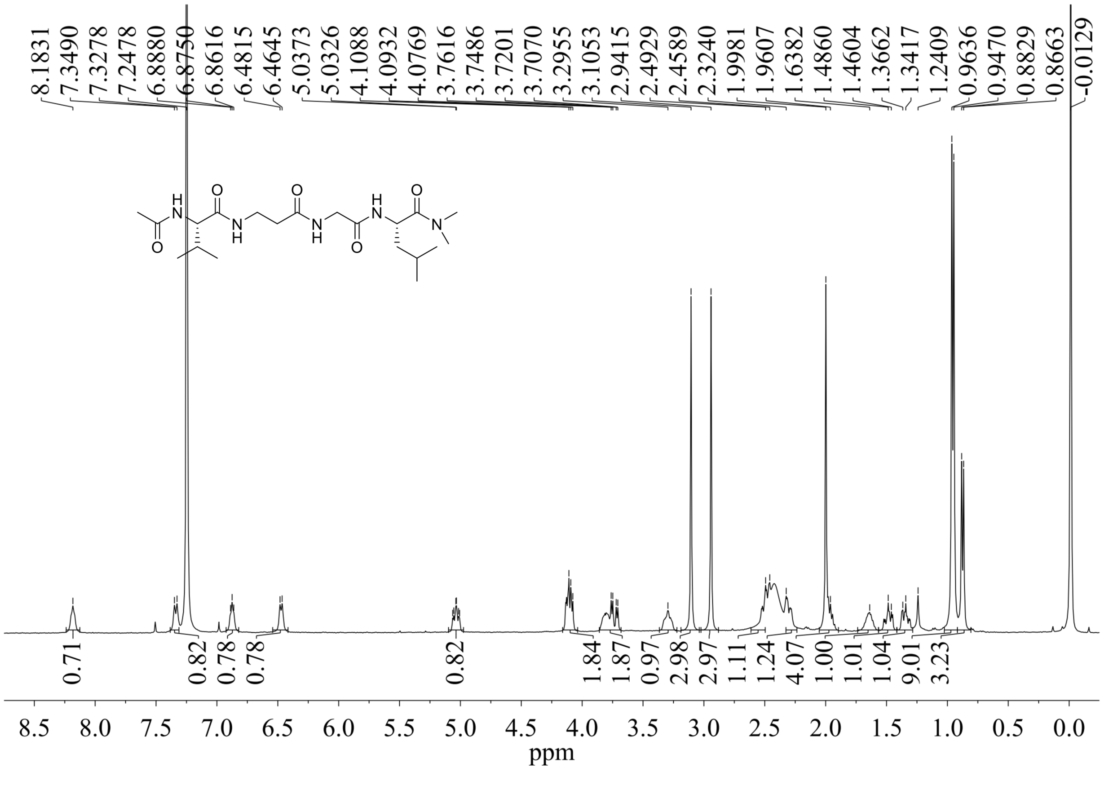

Supplementary Figure 21. ^{1}H NMR spectra of compound **1'** ($CDCl_3$, 298 K, 400 MHz)

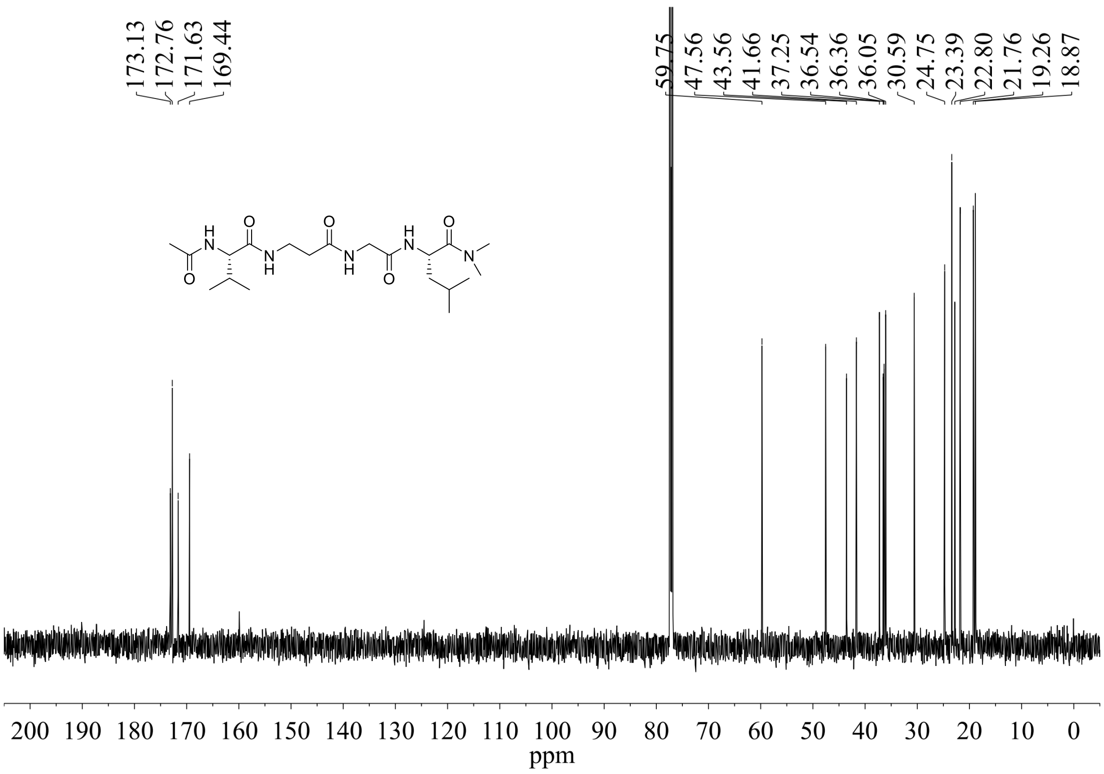

Supplementary Figure 22. ^{13}C NMR spectra of compound **1'** ($CDCl_3$, 299 K, 151 MHz)

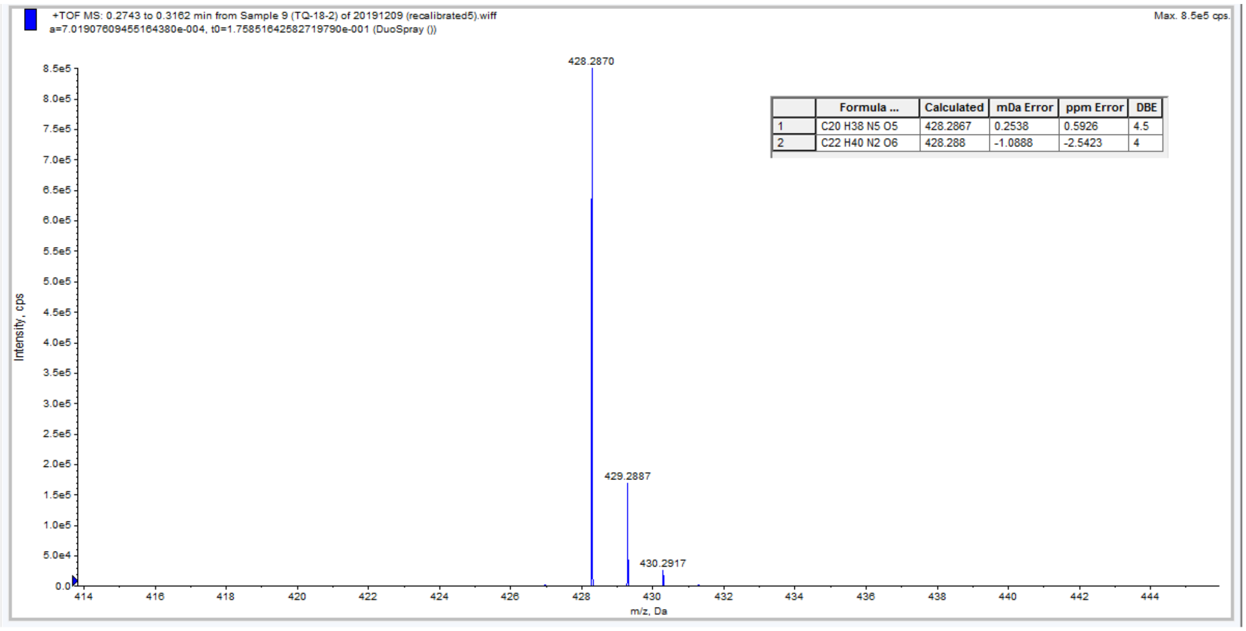

Supplementary Figure 23. MS spectra of compound **1'**

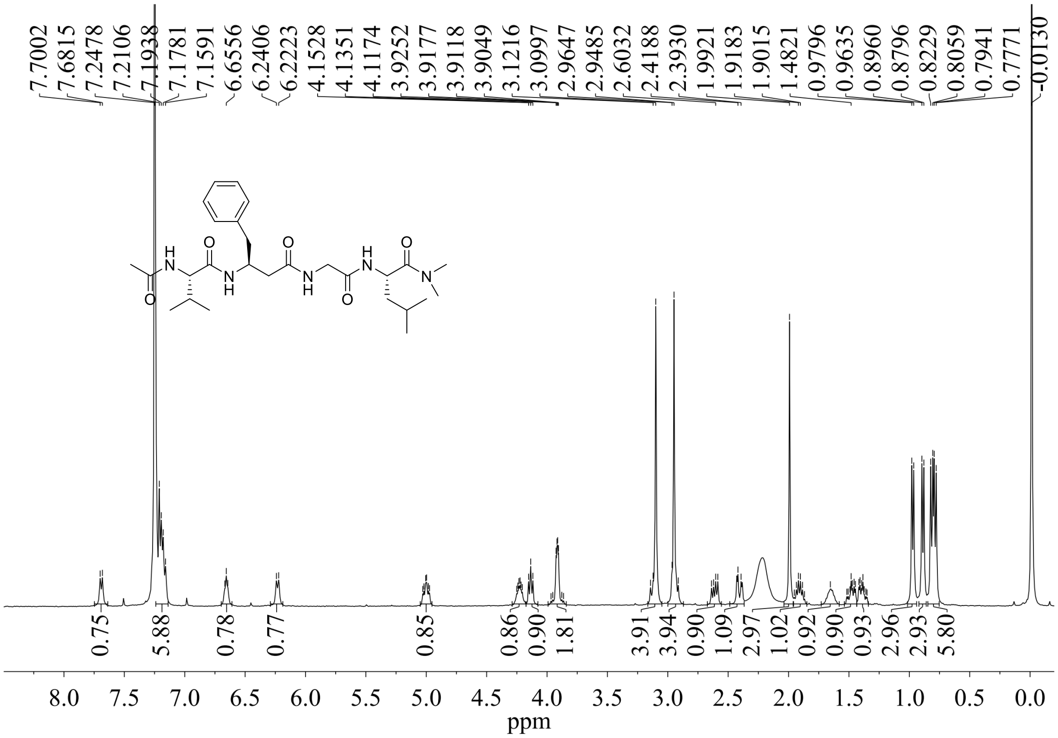

Supplementary Figure 24. ^{1}H NMR spectra of compound **2'** ($CDCl_3$, 298 K, 400 MHz)

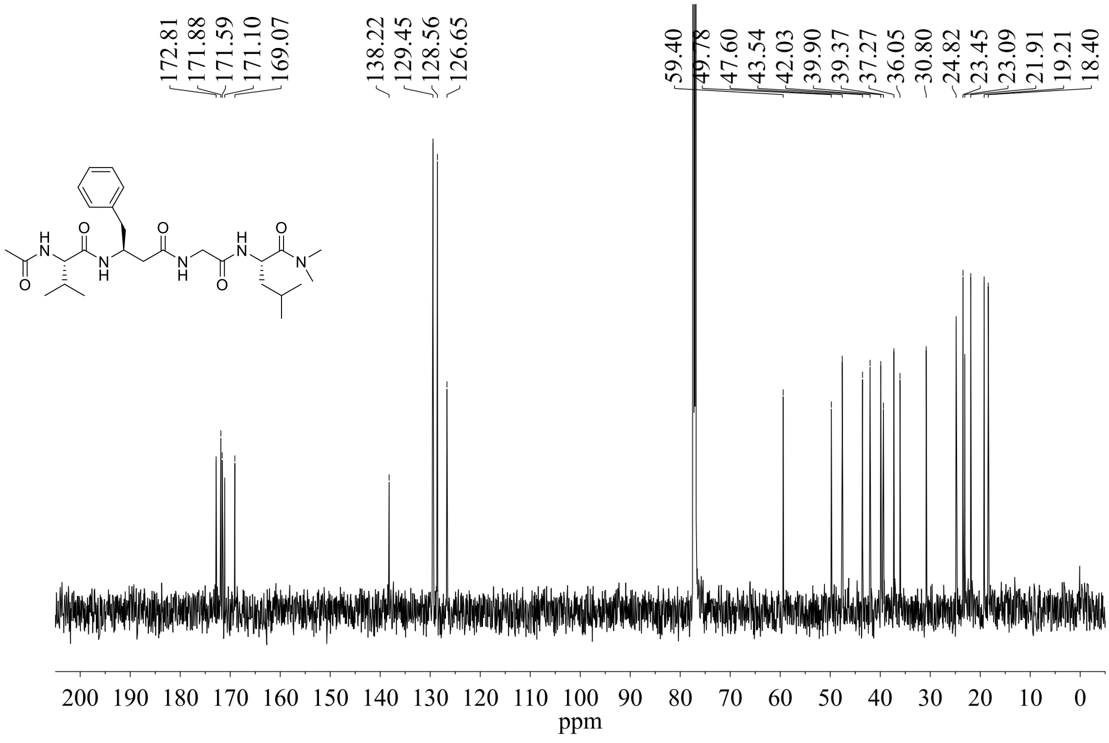

Supplementary Figure 25. ^{13}C NMR spectra of compound **2'** ($CDCl_3$, 299 K, 151 MHz)

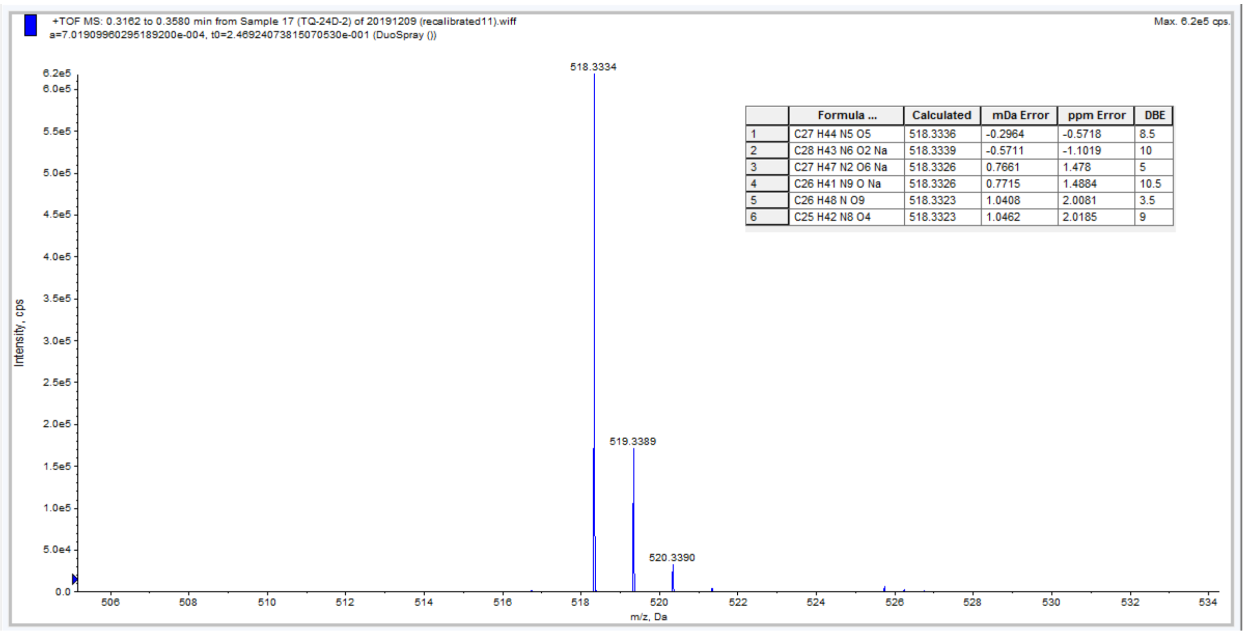

Supplementary Figure 26. MS spectra of compound **2'**

3 Reference

1. Incerti, M.; Russo, S.; Callegari, D.; Pala, D.; Giorgio, C.; Zanotti, I.; Barocelli, E.; Vicini, P.; Vacondio, F.; Rivara, S.; Castelli, R.; Tognolini, M.; Lodola, A., Metadynamics for Perspective Drug Design: Computationally Driven Synthesis of New Protein–Protein Interaction Inhibitors Targeting the EphA2 Receptor. *J. Med. Chem.* **2017,** *60*, 787.

2. Zhang, Y.; Cao, R.; Shen, J.; Detchou, C. S. F.; Zhong, Y.; Wang, H.; Zou, S.; Huang, Q.; Lian, C.; Wang, Q.; Zhu, J.; Gong, B., Hydrogen-Bonded Duplexes with Lengthened Linkers. *Org. Lett.* **2018,** *20*, 1555.

3. Szczepankiewicz, B. G.; Liu, G.; Jae, H.-S.; Tasker, A. S.; Gunawardana, I. W.; von Geldern, T. W.; Gwaltney, S. L.; Wu-Wong, J. R.; Gehrke, L.; Chiou, W. J.; Credo, R. B.; Alder, J. D.; Nukkala, M. A.; Zielinski, N. A.; Jarvis, K.; Mollison, K. W.; Frost, D. J.; Bauch, J. L.; Hui, Y. H.; Claiborne, A. K.; Li, Q.; Rosenberg, S. H., New Antimitotic Agents with Activity in Multi-Drug-Resistant Cell Lines and in Vivo Efficacy in Murine Tumor Models. *J. Med. Chem.* **2001,** *44*, 4416.
